# PD-1 signaling affects cristae morphology and leads to mitochondrial dysfunction in human CD8^+^ T lymphocytes

**DOI:** 10.1186/s40425-019-0628-7

**Published:** 2019-06-13

**Authors:** Jesús Ogando, María Eugenia Sáez, Javier Santos, Cristina Nuevo-Tapioles, Marta Gut, Anna Esteve-Codina, Simon Heath, Antonio González-Pérez, José M. Cuezva, Rosa Ana Lacalle, Santos Mañes

**Affiliations:** 1 Department of Immunology and Oncology, Centro Nacional de Biotecnología (CNB/CSIC), Madrid, Spain; 2 Centro Andaluz de Estudios Bioinformáticos (CAEBi), Seville, Spain; 3 Centro de Biología Molecular-Severo Ochoa (CBMSO/CSIC) and Centro de Investigación Biomédica en Red de Enfermedades Raras (CIBERER-ISCIII), Universidad Autónoma de Madrid, Madrid, Spain; 4 CNAG-CRG, Centre for Genomic Regulation, Barcelona and Institute of Science and Technology (BIST), Barcelona, Spain; 5 Universitat Pompeu Fabra, Barcelona, Spain

**Keywords:** PD-1, CD8, RNA-seq, Metabolism, Mitochondrion, Cristae, MICOS

## Abstract

**Background:**

Binding of the programmed death-1 (PD-1) receptor to its ligands (PD-L1/2) transduces inhibitory signals that promote exhaustion of activated T cells. Blockade of the PD-1 pathway is widely used for cancer treatment, yet the inhibitory signals transduced by PD-1 in T cells remain elusive.

**Methods:**

Expression profiles of human CD8^+^ T cells in resting, activated (CD3 + CD28) and PD-1-stimulated cells (CD3 + CD28 + PD-L1-Fc) conditions were evaluated by RNA-seq. Bioinformatic analyses were used to identify signaling pathways differentially regulated in PD-1-stimulated cells. Metabolic analyses were performed with SeaHorse technology, and mitochondrial ultrastructure was determined by transmission electron microscopy. PD-1-regulated mitochondrial genes were silenced using short-hairpin RNA in primary cells. Blue native gel electrophoresis was used to determine respiratory supercomplex assembly.

**Results:**

PD-1 engagement in human CD8^+^ T cells triggers a specific, progressive genetic program different from that found in resting cells. Gene ontology identified metabolic processes, including glycolysis and oxidative phosphorylation (OXPHOS), as the main pathways targeted by PD-1. We observed severe functional and structural alterations in the mitochondria of PD-1-stimulated cells, including a reduction in the number and length of mitochondrial cristae. These cristae alterations were associated with reduced expression of CHCHD3 and CHCHD10, two proteins that form part of the mitochondrial contact site and cristae organizing system (MICOS). Although PD-1-stimulated cells showed severe cristae alterations, assembly of respiratory supercomplexes was unexpectedly greater in these cells than in activated T cells. CHCHD3 silencing in primary CD8^+^ T cells recapitulated some effects induced by PD-1 stimulation, including reduced mitochondrial polarization and interferon-γ production following T cell activation with anti-CD3 and -CD28 activating antibodies.

**Conclusions:**

Our results suggest that mitochondria are the main targets of PD-1 inhibitory activity. PD-1 reprograms CD8^+^ T cell metabolism for efficient use of fatty acid oxidation; this mitochondrial phenotype might explain the long-lived phenotype of PD-1-engaged T cells.

**Electronic supplementary material:**

The online version of this article (10.1186/s40425-019-0628-7) contains supplementary material, which is available to authorized users.

## Background

Programmed death-1 (PD-1; CD279) acts as a negative regulator of the immune response at the effector phase. PD-1 transmits inhibitory signals in T cells after interaction with its ligands, PD-L1 (B7-H1; CD274) and PD-L2 (B7-DC; CD273). The PD-1/PD-L1/2 system is central to the maintenance of peripheral tolerance by preventing the activation of autoreactive T cells that escape from central tolerance-mediated deletion [1]. High PD-L1/2 levels in non-hematopoietic tissues are associated with suppression of tissue-reactive T cells [2].

Chronic exposure to antigen, as occurs in some infections and most cancers, results in progressive loss of antigen-specific T cell effector capacity, a phenomenon termed exhaustion [3]. Exhausted T cells are characterized by the expression of inhibitory receptors including PD-1. An inverse correlation was reported between T cell function and PD-1 expression levels [4], which has been exploited therapeutically. Immunotherapy based on antibodies that neutralize PD-1 or its ligand PD-L1 effectively restores exhausted T cell-mediated anti-tumor responses in a variety of advanced cancers in humans, with durable effects and high efficacy compared with standard cancer treatments [5].

Despite extensive clinical use of PD-1-based therapeutics, little is known of the mechanisms that underlie PD-1-induced T cell exhaustion. PD-1-mediated inhibition relies on the immunoreceptor tyrosine-based inhibition motif (ITIM) and the immunoreceptor tyrosine-based switch motif (ITSM) in the PD-1 cytoplasmic tail [6]. PD-1 binding to its ligands leads to tyrosine phosphorylation of a residue in its ITSM, which acts as a docking site for recruitment of the Src homology region 2 domain-containing phosphatase-2 (SHP-2, encoded by the *PTPN11* gene). PD-1 can also recruit the tyrosine phosphatase SHP-1 (encoded by the *PTPN6* gene), but only SHP-2 colocalizes with PD-1 and the TCR at the immune synapse [7]. SHP-2 recruitment to activated PD-1 is postulated to cause dephosphorylation of TCR-induced signaling intermediates such as ZAP70 [6, 7]. Regardless of its tyrosine phosphatase activity, SHP-2 positively regulates various signaling cascades [8, 9], including extracellular signal-regulated kinase (ERK) activation following TCR triggering [10, 11]. A recent report showed that SHP-2 is totally dispensable for PD-1 signaling and T cell exhaustion in vivo [12].

PD-1 also targets metabolic reprogramming in CD4^+^ and CD8^+^ T cells. Resting and memory T cells typically use an oxidative metabolic program (OXPHOS) characterized by increased mitochondrial fatty acid oxidation and spare respiratory capacity (SRC) [13, 14]. In contrast, effector T cells rewire their metabolism to potentiate aerobic glycolysis, which triggers proliferation and expression of effector cytokines such as interferon-gamma (IFNγ). Mitochondrial function and integrity are nonetheless critical for both effector and memory phases of T cell differentiation [15].

In vitro studies show that PD-1 stimulation reduces the extracellular acidification rate (ECAR) as well as basal and stimulated O_2_ consumption rates (OCR), which indicates that PD-1 engagement dysregulates both glycolytic and mitochondrial energetics in activated T cells [16]. Similar metabolic alterations are observed in vivo in exhausted virus-reactive and tumor-infiltrating lymphocytes (TIL) [17–19]. Whereas PD-1-mediated suppression of glycolysis might be caused by abrogation of the AKT and mTOR pathways downstream of the TCR [16, 20], the mechanisms by which PD-1 affects mitochondria are mainly unknown.

To investigate the PD-1-elicited signaling pathways that cause T cell dysregulation, we analyzed the expression profiling of human CD8^+^ T cells in conditions that mimic simultaneous engagement of PD-1 and the TCR/CD3 complex. We show here that PD-1 engagement triggers a specific, time-dependent genetic program different from that in resting cells. This finding suggests that in addition to blocking TCR-mediated signals, PD-1 can generate specific signaling pathways that dysregulate T cell function. We provide a mechanistic framework that explains the reduction in the number and length of mitochondrial cristae in PD-1-engaged cells, which involves reduced expression of two proteins that form part of the MICOS complex.

## Methods

For a more detailed description, see Additional file 1.

### Cell culture and T cell activation

Human embryonic kidney (HEK)-293 T cells (ATCC) were cultured in DMEM (BioWest). Peripheral blood mononuclear cells (PBMC) were obtained from buffy coats from healthy donors (Centro de Transfusiones of the Comunidad de Madrid, Spain), using Ficoll density gradients. CD8^+^ T cells were isolated by negative selection (EasySep human CD8^+^ T cell, Stem Cell Technologies; 86–95.5% purity), and cultured in RPMI-1640 medium (BioWest).

For activation, CD8^+^ T cells were incubated (1:3.5 ratio) with tosyl-activated magnetic beads (Dynabeads M-450; Thermo Scientific) coated with 8% anti-CD3 (HIT3a, BD Biosciences), 10% anti-CD28 (CD28.2, BioLegend), and 82% control IgG_1_ (T_ACT_), or with anti-CD3, anti-CD28, and 82% PD-L1-Fc chimeric protein (R&D Systems) (T_ACT + PD1_); IgG_1_-coated beads were used as control (T_CTRL_). In some experiments, PD-L1-Fc was used at 16.4, 3.3% or 0.66%. In some experiments, CD8^+^ T cells were incubated with T_ACT + PD1_ beads (48 h, 37 °C), which were mechanically released, removed with a magnet, and the cells restimulated with T_ACT_ or T_CTRL_ beads (48 h, 37 °C). As positive control, naïve CD8^+^ T cells were incubated (48 h, 37 °C) with plate-bound anti-CD3 (5 μg/ml; UCHT1, BD Biosciences) and soluble anti-CD28 (2 μg/ml) antibodies.

T cell activation was confirmed by FACS (Cytomics FC500 or Gallios cytometers; Beckman Coulter) using anti-CD25-PE (B1.49.9, Beckman-Coulter), −CD279-APC (MIH4, eBioscience), −CD69-PCy5 (TP1.553, Inmunotech), and -CD8-FITC (B9.11, Beckman-Coulter) antibodies. IFNγ was detected by intracellular staining using anti-IFNγ-PE (B27, Pharmingen) antibody in permeabilized cells (Beckman-Coulter) pretreated with brefeldin A (10 μg/ml, 4 h, 37 °C; eBioscience). Dead cells were detected with propidium iodide (2.5 μg/test, 1 min), or the LIVE/DEAD stain kit (Invitrogen). Appropriate isotypes were used as negative controls. Data were analyzed using Kaluza and FlowJo software.

hCD8^+^ T cell proliferation was determined by [methyl-^3^H] thymidine (1 μCi/well; Perkin Elmer) incorporation into DNA, in a 1450 Microbeta liquid scintillation counter (Perkin Elmer).

### RNA-seq analysis

The RNA-seq libraries were prepared using an Illumina TruSeq Stranded Total RNA Sample Preparation kit (Illumina). Library size and quality was assessed in an Agilent DNA 7500 Bioanalyzer assay (Agilent). Each library was sequenced using TruSeq SBS Kit v3-HS, in paired end mode with read length 2 × 76 bp. On average, we generated 36 million paired-end reads for each sample in a fraction of a sequencing lane on HiSeq2000 (Illumina). Image analysis, base calling, and quality scoring of the run were processed by Real Time Analysis (RTA 1.13.48) software, followed by generation of FASTQ sequence files by CASAVA 1.8.

RNA-seq reads were aligned with the human reference genome (gencode v19) using the GEMtools RNA-seq pipeline v1.7 (http://gemtools.github.io), which is based on the GEM mapper [21]. Expression quantification at the gene level was calculated with Flux (http://sammeth.net/confluence/display/FLUX/Home). RNA-seq data were analyzed using the DESeq2 R Bioconductor package [22]. Raw counts of sequencing reads were normalized to the effective library size. Real-time quantitative PCR (qPCR) was performed in an ABI PRISM7900HT system (Applied Biosystems) with indicated primers (Additional file 2: Table S1).

A likelihood ratio test (LRT) was used to test for differences over multiple time points. This test compares a full model, including an interaction term class:time, with a reduced model without the interaction term; this permits to determine whether PD-1 treatment induces change of a specific gene at any point after time 0. This class-specific effect is measured as a *p* value for interaction (p_inter_) and FC values for T_ACT + PD1_ vs. T_ACT_ cells at each time point. Genes with a significant p_inter_ were analyzed by STEM (Short Time-series Expression Miner) software [23] for cluster analysis and integration with the Gene Ontology (GO) database (http://geneontology.org/). These genes were analyzed for enrichment in KEGG signaling pathways using the online tool Webgestalt (http://www.webgestalt.org). Genes involved in metabolic pathways (KEGG hsa011000) were further explored for known interactions using Cytoescape (http://www.cytoscape.org/). GO enrichment analysis was performed using BINGO. GO categories were summarized and visualized using ClueGO or REVIGO.

### Metabolic assays

Cellular oxygen consumption (OCR) and extracellular acidification rates (ECAR) were determined in Seahorse XF Base Medium supplemented with 25 mM glucose (Sigma-Aldrich), 2 mM L-glutamine and 1 mM sodium pyruvate (both from BioWest) using the XF cell Mito Stress Kit (SeaHorse Bioscience), in an XF24 Extracellular Flux Analyzer (SeaHorse Bioscience; Agilent Technologies). Fatty acid oxidation (FAO) was determined in Krebs-Henseleit buffer (KHB) supplemented with 0.5 mM carnitine (Sigma-Aldrich) and 2.5 mM glucose, using palmitate as substrate, in the Agilent Seahorse XF96 Extracellular Flux Analyzer.

Lactate levels were determined enzymatically in extracts from T_CTRL_, T_ACT_ and T_ACT + PD1_ cells after 48 h stimulation, using a fluorometric lactate assay kit (Cell Biolabs) according to supplier’s protocol; fluorescence was quantified in a Filter Max F5 microplate reader (Molecular Devices) at 530/590 nm excitation/emission. A lactate standard curve was generated in all assays and used to extrapolate relative fluorescent units (RFU) measured in the samples.

### Blue native and immunoblot analyses

Equal amounts of Triton X-100-based cells lysed were analyzed by SDS-PAGE analysis and immunoblotted with specific antibodies (see Additional file 1) [24]. For blue native analyses, we obtained a mitochondria-enriched fraction by cell lysis with hypotonic buffer and homogenization with a polypropylene pestle homogenizer. Nuclei and unbroken cells were removed, and mitochondria obtained by centrifugation (12,000×*g*) from the cytosolic fraction. The enriched mitochondrial fraction was suspended in 50 mM Tris-HCl pH 7.0 containing 1 M 6-aminohexanoic acid, lysed in 10% digitonin at 4 g/g mitochondrial proteins, and mitochondrial proteins fractionated in blue native gels.

### Functional and structural mitochondria studies

Total mitochondrial mass, mitochondrial membrane potential (ΔΨ*m*) and reactive oxygen species (ROS) were determined by FACS using MitotrackerGreen FM, tetramethylrhodamine, methyl ester (TMRM) and MitoSOX probes (Thermo Fisher), respectively. DNP (2,4-dinitrophenol) was used as ΔΨ*m* negative control. Dead cells were excluded by diamino-2-phenylindol (DAPI) staining. Mitochondrial DNA (mtDNA) was extracted from hCD8^+^ cells with the DNeasy Blood and Tissue kit (Qiagen) and quantified by RT-qPCR using primers for MT-TL1 tRNA (Leu)(UUR) [25]; the α2-microglobulin gene was used for normalization.

Immunofluorescence analyses were performed in paraformaldehyde-fixed CD8^+^ T cells, permeabilized with Triton X-100 (0.1%). After blocking, cells were stained sequentially with anti-human aconitase-2 (6F12BD9, Abcam) and goat anti-mouse Alexa 488 (Molecular Probes). Samples were mounted in Prolong Gold Antifade Reagent with DAPI (Cell Signaling) and images captured in a Leica Microsystems microscope (LAS X v2.01; 60x objective). Mitochondrial morphology was determined with ImageJ [26].

For transmission electron microscopy, fixed cells were treated sequentially with 1% osmium tetroxide (TAAB Laboratories) and 2% aqueous uranyl acetate, dehydrated with acetone, embedded in EPON 812 resin, and polymerized. Ultrathin sections (70 nm-thick; Ultracut EM UC6, Leica Microsystems) in 200 mesh nickel EM grids (Gilder) were stained with 3% aqueous uranyl acetate and lead citrate, and analyzed on a JEOL JEM 1011 electron microscope. The number of mitochondria per cell and cristae length were quantified by two independent observers blind to the experiment.

### CHCHD3 silencing experiments

Lentiviruses encoding CHCHD3 or control short hairpin RNA (shRNA; Genecopoeia) were produced in HEK-293 T cells. Prior to transduction, hCD8^+^ cells were stimulated with anti-CD3- and -CD28 antibody-coated beads, then transduced with viral supernatants at 10–20 m.o.i in the presence of polybrene. CHCHD3 silencing was determined by qPCR and by immunoblot.

### Statistical analysis

Normal or parametric distribution of the data was analyzed. For comparison between two conditions, data were analyzed with the Mann-Whitney U test. For multiple non-parametric comparisons, Kruskal-Wallis followed by Dunn’s post-test was used. For multiple parametric comparisons, data were analyzed by one- or two-way ANOVA with the Bonferroni post-hoc test. For the same samples with different treatments, paired Student’s *t*-test was performed for two comparisons or paired repeated-measures one-way ANOVA for more than two conditions. Differences were considered significant when *p* < 0.05. All statistical analyses were performed using Prism 7.0 software (GraphPad).

## Results

### RNA-seq distinguishes specific PD-1-induced gene sets in human CD8^+^ T cells

**Fig. 1:**
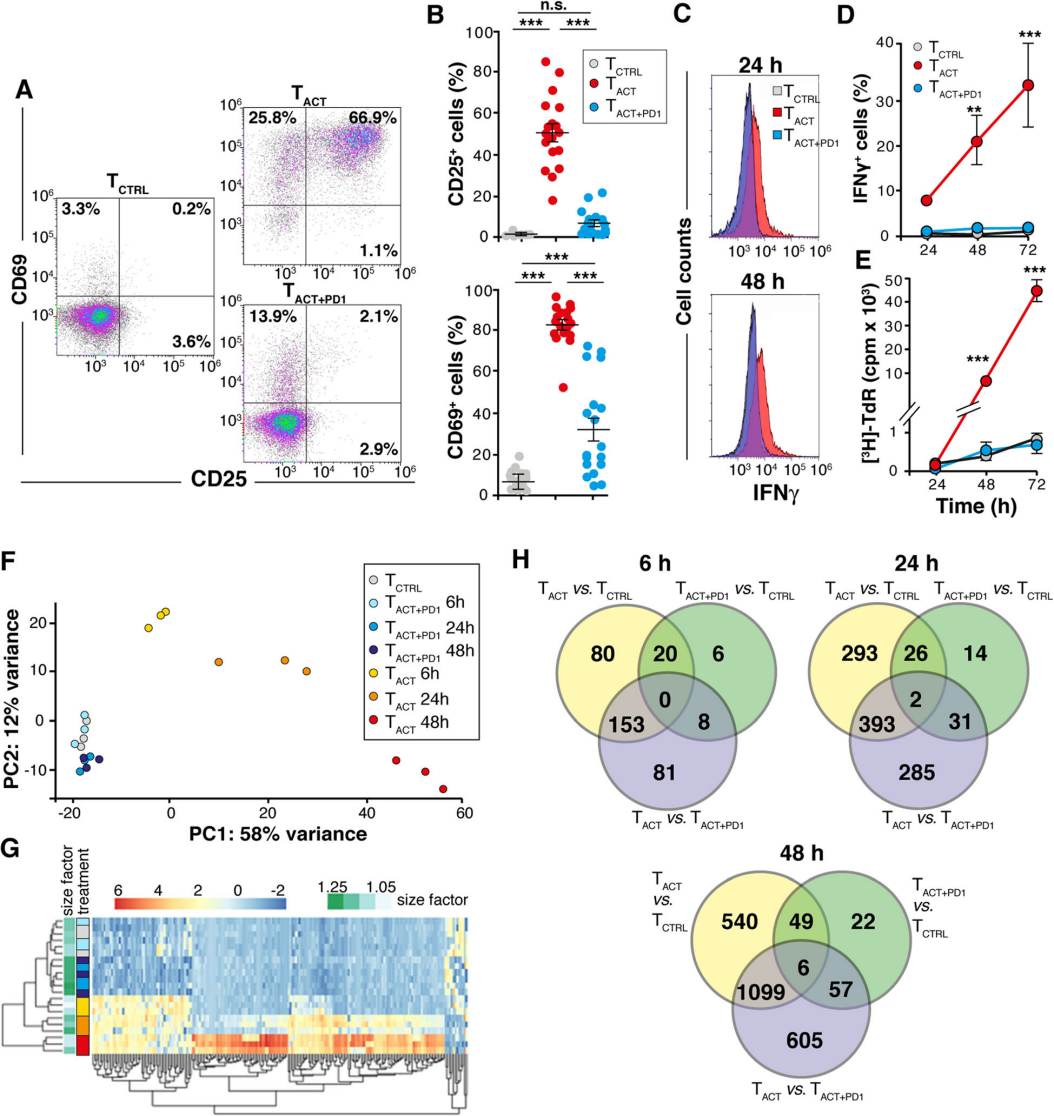
Characterization of gene expression profiles in CD8^+^ T cells after PD-1 ligation. **a** Representative dot plots showing CD25 and CD69 staining of primary human CD8^+^ T cells after 48 h stimulation with T_CTRL_, T_ACT_, and T_ACT + PD1_ beads. **b** Quantification of CD25- and CD69-expressing cells from dot plots as in **a**. Each dot represents a donor (*n* = 18). **c** Representative histograms showing IFNγ production by cells stimulated as in **a** for 24 and 48 h. **d** Quantification of data from **c** (*n* = 4). **e** Thymidine ([^3^H]-TdR) incorporation by cells stimulated as in **a** (*n* = 5). **f** PCA plot using the rlog-transformed values from the RNA-seq analysis. Each unique combination of cell stimulation and time is assigned a distinct color. **g** Hierarchical clustering dendrogram of the top 200 most-variable genes across samples. The heat map color code (left) uses the combination of cell stimulation/time as in **f**. **h** Venn diagrams showing the number of differentially expressed genes between indicated conditions at different times. For **d** and **e**, data show mean ± SEM. ** *p* < 0.01; *** *p* < 0.001, one-way (**b**) or two-way ANOVA (**d**, **e**) with Bonferroni post-test

To determine how PD-1 signals change gene expression during activation of human (h)CD8^+^ T cells, we used an in vitro system that mimics the simultaneous engagement of PD-1 and the TCR/CD3 complex. Purified hCD8^+^ T cells were stimulated with magnetic beads conjugated with stimulating anti-CD3 and -CD28 antibodies (T_ACT_ cells), or with anti-CD3, anti-CD28, and PD-L1-Ig fusion protein (T_ACT + PD1_ cells); hCD8^+^ T cells incubated 6 h with polyclonal IgG-conjugated beads were used as control (T_CTRL_ cells). In these conditions, PD-1 consistently inhibited hCD8^+^ T cell activation and effector functions, determined by a reduction in CD25, CD69 and IFNγ expression (Fig. 1a-d), as well as decreased proliferation (Fig. 1e). The PD-1-induced reduction was dose-dependent (Additional file 3: Figure S1).

Total RNA was isolated from T_CTRL_, T_ACT_ and T_ACT + PD1_ at 6, 24 and 48 h post-stimulation, and gene expression analyzed by RNAseq. MA plots representing log2-fold changes (FC) against mean normalized counts were generated for all experimental conditions (Additional file 4: Figure S2; red dots indicate significant genes with a 10% false discovery rate (FDR)). Principal components analysis (PCA; Fig. 1f) and hierarchical clustering of the top 200 most-variable genes across samples (Fig. 1g) were used to determine similarity between expression profiles. These analyses clustered the three biological replicates of T_ACT_ cells at each time analyzed; these analyses also differentiated T_ACT + PD1_ samples after 24 and 48 h stimulation from the T_CTRL_ and T_ACT + PD1_ after 6 h stimulation, which were very close or intermixed. Venn diagrams showed a number of unique sets of differentially expressed genes in the T_ACT + PD1_ cells compared to T_CTRL_ and T_ACT_ counterparts (Fig. 1h). These results suggest that PD-1 engagement not only prevented hCD8^+^ T cell activation, but also triggered a specific transcriptional program in hCD8^+^ T cells.

### PD-1 engagement impairs expression of metabolic genes in human CD8^+^ T cells

**Fig. 2:**
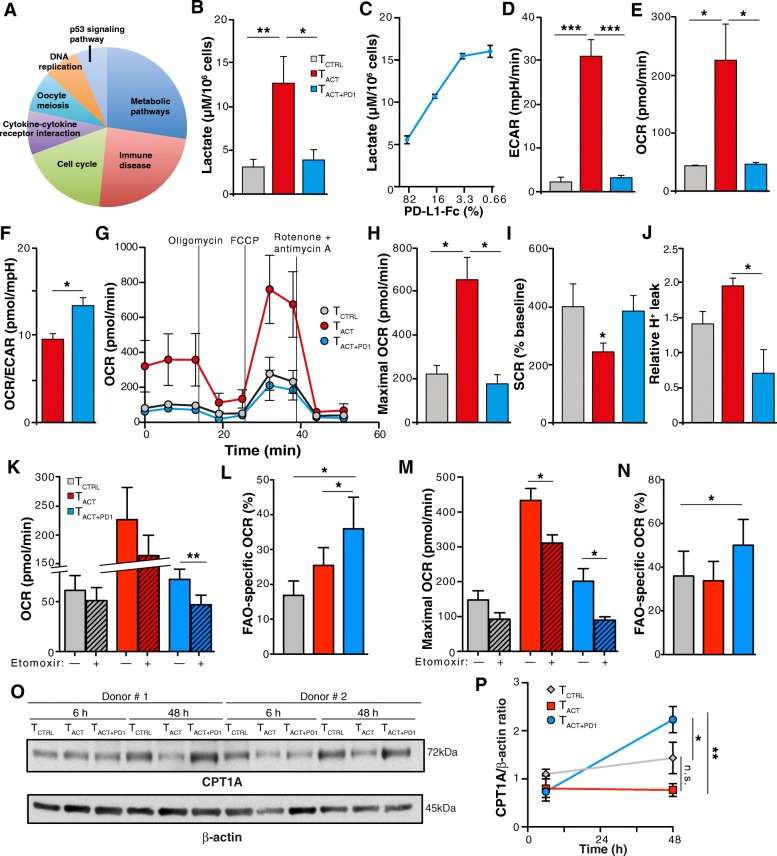
PD-1 ligation impairs mainly CD8^+^ T cell metabolism. **a** KEGG signaling pathways with the highest scores significantly enriched in the 578 transcripts selected by LRT. **b** Lactate production in hCD8^+^ T cells stimulated 48 h with T_CTRL_, T_ACT_ and T_ACT + PD1_ beads. **c** Lactate production in hCD8+ T cells stimulated 48 h with T_ACT + PD1_ beads containing the indicated amounts of PD-L1-Fc. **d-f** hCD8^+^ T cells were stimulated with beads as in **b** and analyzed with SeaHorse. Basal extracellular acidification rate (ECAR; D), basal O_2_ consumption rate (OCR; **e**), and basal OCR/ECAR ratio (**f**). **g** OCR obtained during mitochondrial stress test in cells stimulated as in **b**, performed by injection of oligomycin, the mitochondrial uncoupler FCCP, and the electron transport chain inhibitors antimycin A/rotenone. **h-j** Maximal OCR obtained after FCCP injection (**h**), spare respiratory capacity (SRC; **i**) calculated as the difference between maximal and basal OCR, and relative proton leak (**j**) determined as OCR after oligomycin and subsequent injection of rotenone plus antimycin A. **k-n** hCD8^+^ T cells were stimulated with beads as in **b**, treated with etomoxir or vehicle and analyzed with SeaHorse, using palmitate as substrate. Basal OCR with vehicle (solid) or with etomoxir (hatched) (**k**), FAO-specific OCR from data in **k** (**l**), maximal OCR after FCCP injection in vehicle or etomoxir-treated cells (**m**), FAO-specific maximal OCR calculated from **m** (**n**). **o** Representative immunoblots for CPT1A and β-actin (loading control) in CD8^+^ T cells stimulated as indicated. **p** Densitometric analysis of immunoblots as in **o**. The CPT1A/β-actin ratio is shown, with the value for T_CTRL_ cells as reference (*n* = 3 donors). Data are mean ± SEM from six (**b**, **d**-**j**), four (**k**-**n**) or three (**p**) donors; for **c**, data are mean ± SD representative of one donor from two. ** *p* < 0.01, * *p* < 0.05, Kruskal-Wallis with Dunn’s post-hoc test for multiple comparisons (**b**, **d**-**f**, **h**-**k**, **m**), two-tailed Student’s *t*-test (**l**, **n**), or two-way ANOVA with Newman-Keuls post-hoc test for multiple comparisons (**p**)

We used LRT to identify genes expressed differentially over time. This type of analysis identifies genetic patterns impaired by PD-1 engagement more reliably than direct comparison between T_CTRL_, T_ACT_ and T_ACT + PD1_ RNAseq data at each time point. LRT analysis identified 1651 genes with divergent expression between T_ACT_ and T_ACT + PD1_ (p_inter_ < 0.05), but only 578 passed FDR correction (Adj-p_inter_ < 0.05); Additional file 5: Table S2 shows the top 20 genes in this analysis. KEGG pathway analysis using these 578 genes indicated that, in addition to pathways related to cell cycle and immune function, there was significant enrichment in metabolic genes, with 43 genes in this category (Fig. 2a; Additional file 6: Table S3). The primary metabolic processes with the most differentially regulated genes were amino acid, nucleotide and carbohydrate (glycolysis and the pentose phosphate) metabolism, the citrate cycle and OXPHOS (Additional file 7: Figure S3).

GO enrichment analysis of the 43 metabolic genes showed generation of precursor metabolites and energy and oxidative phosphorylation among the most represented biological processes (Additional file 8: Figure S4); the most represented molecular functions were NADH dehydrogenase and oxidoreductase activities (Additional file 9: Figure S5). Mitochondria and the respiratory chain were also identified as significantly enriched cellular components (Additional file 10: Figure S6).

### PD-1 engagement suppresses glycolysis and oxidative phosphorylation in CD8^+^ T cells

To validate the transcriptional changes with metabolic alterations, we focused on glycolysis and OXPHOS, key metabolic pathways for T cell differentiation and function [13, 27]. We found that lactate production, a glycolysis indicator, was reduced in T_ACT + PD1_ compared with T_ACT_ cells, in a dose-dependent manner (Fig. 2b, c). T_ACT + PD1_ cells similarly showed a significant ECAR reduction (Fig. 2d), which suggested that PD-1 ligation effectively inhibited the glycolytic pathway in CD8^+^ T cells. When we used high glucose levels as an energy source, basal OCR, an OXPHOS indicator, was significantly higher in T_ACT_ than in T_CTRL_ and T_ACT + PD1_ cells (Fig. 2e); the OCR/ECAR ratio was nonetheless higher in T_ACT + PD1_ than in T_ACT_ cells (Fig. 2f), which suggested that T_ACT + PD1_ cells preferentially use OXPHOS rather than glycolysis to generate ATP.

To analyze additional parameters of mitochondrial metabolism, we measured OCR in real time in basal conditions and after addition of several mitochondrial inhibitors (Fig. 2g). Addition of FCCP, which uncouples ATP synthesis from the electron transport chain, showed that maximal respiration capacity was higher in T_ACT_ than in T_CTRL_ and T_ACT + PD1_ cells (Fig. 2h). T_CTRL_ and T_ACT + PD1_ cells nonetheless had a substantial mitochondrial SRC, as indicated by the difference between maximal and basal OCR (Fig. 2i). The elevated SRC, a parameter associated with long-term survival [14], and the higher OCR/ECAR ratio suggest more efficient OXPHOS in T_ACT + PD1_ than in T_ACT_ cells. Confirming this idea, proton leak (determined as OCR after oligomycin relative to OCR after rotenone and antimycin A) was significantly lower in T_ACT + PD1_ than in T_ACT_ cells (Fig. 2j); there was also a tendency to lower proton leak in T_ACT + PD1_ than in T_CTRL_ cells (Fig. 2j).

To further study metabolic differences in the mitochondria of PD-1-stimulated cells, we measured OCR using palmitate as a substrate, alone or in the presence of etomoxir, which inhibits carnitine palmitoyltransferase 1A (CPT1A), a central enzyme for long-chain fatty acid oxidation in mitochondria. Etomoxir led to greater inhibition of basal and maximal (after oligomycin and FCCP treatment) OCR in T_ACT + PD1_ than in T_CTRL_ and T_ACT_ cells (Fig. 2k-n), which indicated greater OXPHOS dependence on FAO in T_ACT + PD1_ cells than in the other conditions. We also found time-dependent induction of CPT1A in T_ACT + PD1_ compared to T_CTRL_ and T_ACT_ cells (Fig. 2o, p), which might explain the mechanism underlying the higher FAO capacity of PD-1-stimulated cells. These results indicate that PD-1 signals reprogram CD8^+^ T cell metabolism for efficient use of FAO-dependent mitochondrial OXPHOS, which resembles some aspects of long-lived memory T cells [14]. Moreover, the distinct FAO-dependent OXPHOS between T_CTRL_ and T_ACT + PD1_ cells (Fig. 2l, n) suggests that PD-1-induced metabolic changes are not simply blockade of T cell activation, but involve unique, time-dependent programs induced by PD-1 engagement.

### PD-1 ligation reduces mitochondrial polarization and ROS production

**Fig. 3:**
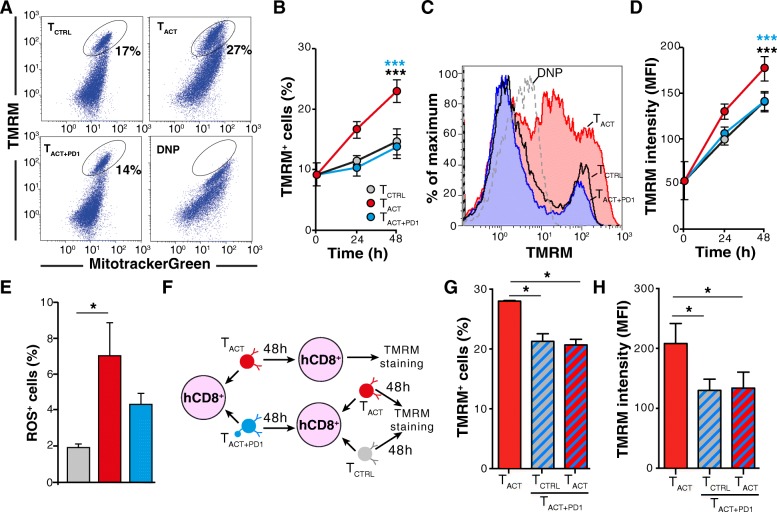
PD-1 inhibits mitochondrial function in activated CD8^+^ T cells. **a** Representative dot plots of CD8^+^ T cells stained with MitoTracker Green and TMRM to determine the effect of the indicated stimuli on mitochondrial polarization. Incubation with the depolarizing agent DNP was used as negative control. **b** Time-dependent expansion of TMRM^+^ cells after indicated stimuli (*n* = 5). **c** Representative histograms of T_CTRL_, T_ACT_ and T_ACT + PD1_ cells after 48 h stimulation. TMRM fluorescence is shown of DNP-treated T_ACT_ cells (negative control; dotted line). **d** Mean fluorescence intensity of TMRM^+^ CD8^+^ T cells at different times post-stimulation, assessed from data as in **c** (*n* = 5). **e** Percentage of ROS^+^ cells as detected with the MitoSOX Red probe. **f** Scheme for analyzing the reversibility of PD-1 effects on mitochondrial potential. **g, h** Percentage of TMRM^+^ cells and TMRM mean fluorescence intensity in T_ACT_ and pre-treated T_ACT + PD1_ cells re-stimulated with T_CTRL_and T_ACT_ beads (*n* = 3). Data shown as mean ± SEM. *** *p* < 0.001, two-way ANOVA with Bonferroni post-hoc test; * *p* < 0.05, two-tailed paired Student’s *t*-test

We analyzed mitochondria bioenergetics in live cells by combining the ΔΨm-sensitive TMRM and the ΔΨm-independent MitotrackerGreen probes; the depolarizing agent DNP was used as a TMRM staining control (Fig. 3a). Compared with T_CTRL_ cells, CD8^+^ T cell activation caused a significant increase in both the number of cells with polarized mitochondria (Fig. 3b) and the TMRM fluorescence bound to these mitochondria (Fig. 3c-d). PD-1 ligation abrogated the ΔΨm increase caused by activation stimuli (Fig. 3c-d). Reactive oxygen species (ROS) production nonetheless did not differ statistically between T_ACT + PD1_ and T_ACT_ cells (Fig. 3e). It appears that although PD-1 affects mitochondrial function, these organelles retain some respiratory capacity compared to that of resting T_CTRL_ cells.

We tested whether the PD-1 effects on ΔΨm were reversible. CD8^+^ T cells were incubated with T_ACT + PD1_ beads and, after PD-L1 washout, stimulated with T_ACT_ or T_CTRL_ beads (Fig. 3f). Re-stimulation of T_ACT + PD1 with_ T_CTRL_ beads indicated that pre-incubation of cells with PD-L1 reduced both the percentage of cells with polarized mitochondria as well as TMRM fluorescence intensity compared to T_ACT_ cells. But more important, after re-stimulation with T_ACT_ beads, the PD-L1-preincubated cells did not recover either the percentage of TMRM^+^ cells or fluorescence intensity to the levels observed in primary T_ACT_ cells (Fig. 3g, h). These results suggest that PD-1 effects on these mitochondrial parameters were irreversible.

### PD-1 controls expression of genes involved in mitochondrial structure and function

Of the 578 genes selected by LRT, 84 coded for transcripts enriched in mitochondrial-related GO categories (Additional file 11: Figure S7). These 84 genes were not only related to metabolic pathways, but also included those involved in mitochondrial DNA replication and repair (*FEN1, TOP2A, XRCC3*), translation (*POP7, MRPL39, MRPS12*), protein import machinery (*TIMM22, TIMM23, TOMM34*), fusion/fission (*MIEF1, MTCH1*), cristae structure and organization (*CHCHD3, CHCHD10, HSPA9*), and assembly of protein complexes of the respiratory chain (*ATP5G1, COX8A, NDUFB3, SELRC1, UQCRC2*) (Additional file 12: Table S4).

**Fig. 4:**
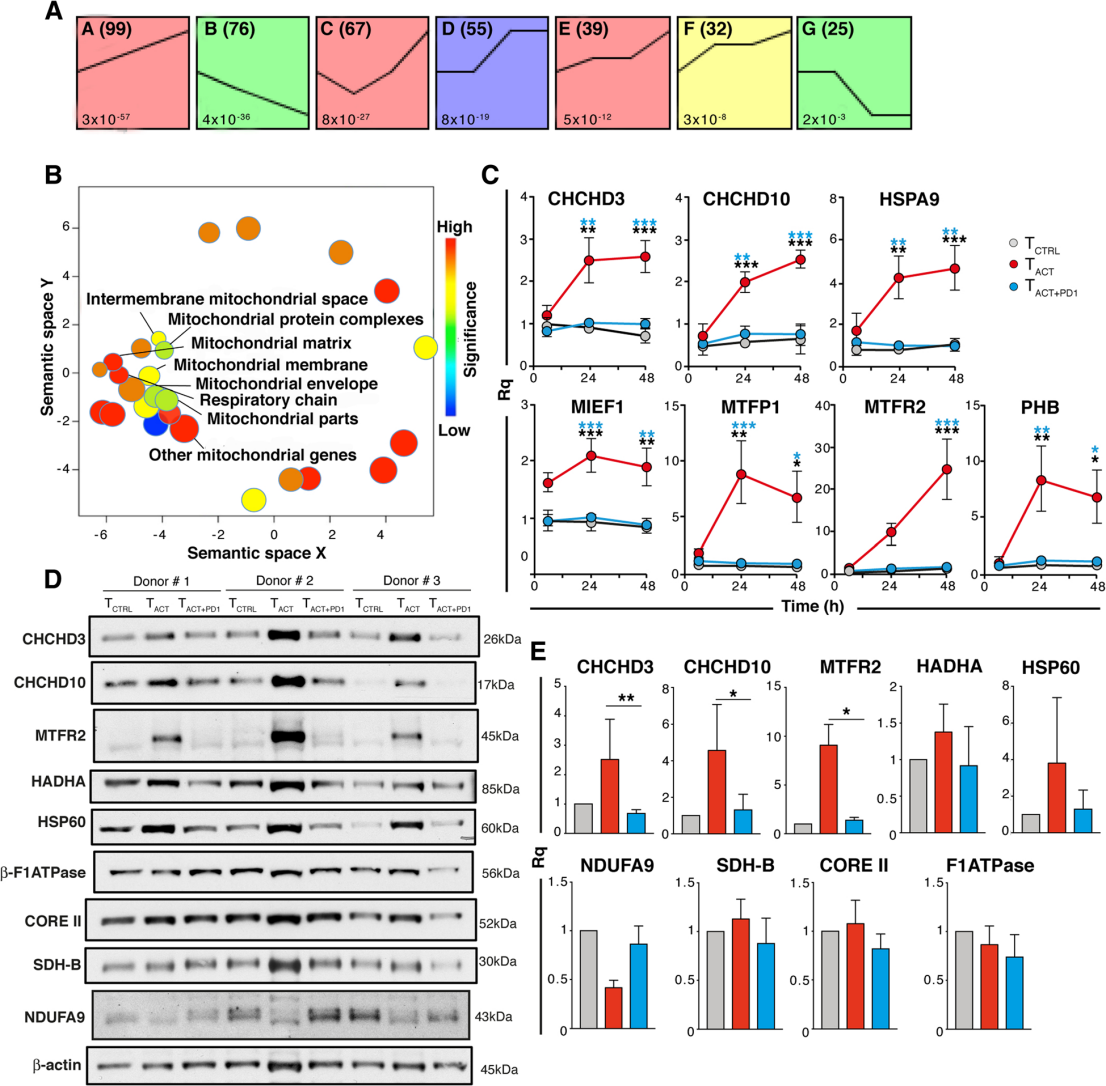
Validation of changes in expression of mitochondrial-related genes after PD-1 ligation. **a** STEM clusters of expression profiles in T_ACT_ and T_ACT + PD1_ cells. Only significant profiles are shown, ordered by *p* value (bottom left). The line in each STEM cluster represents the average temporal expression profile for the genes assigned to the cluster. The number of genes in each profile is indicated (top right). **b** Scatter plot showing GO terms of STEM profiles A, E and F, represented as circles and clustered according to semantic similarities as determined by REViGO. Circle area is proportional to the significance of GO term overrepresentation; color indicates the log_10_ of the corrected *p* value for enrichment. **c** Time-course variation of the relative quantity (Rq) of indicated transcripts in T_CTRL_, T_ACT_, and T_ACT + PD1_ cells isolated from independent donors (*n* ≥ 3). **d** Representative immunoblots for proteins in CD8^+^ T cells stimulated as indicated (*n* ≥ 3 donors). **e** Densitometric analysis of immunoblots as in **d**. The Rq was calculated as the ratio between each protein and β-actin, taking the value for T_CTRL_ cells as reference. For C and E, data are mean ± SEM. * *p* < 0.05, ** *p* < 0.01, *** *p* < 0.001, using two-way ANOVA with Bonferroni post-test (**c**) or Kruskal-Wallis with Dunn’s post-hoc test for multiple comparisons (**e**); only significant differences are indicated

We used STEM software [23] to analyze and cluster our gene expression dataset more stringently. STEM clustering of logFC values generated eight model expression profiles significantly enriched (FDR < 0.05) for transcripts expressed longitudinally in T_ACT_ vs T_ACT + PD1_ cells (Fig. 4a). Profile A, which clustered transcripts whose expression increased over time in T_ACT_ compared to T_ACT + PD1_ cells, was specifically enriched for genes in *Mitochondrial Protein Complex* (including *ATP5G1, CHCHD3, COX8A, DNA2, NDUFAB1, NDUFB3, NDUFB7, PPIF, TIMM22, TIMM23, TOMM40, TOMM40L* and *UQCRC2*), as well as in 27 transcripts of other mitochondrial-related profiles (Fig. 4b). This finding suggests that genes involved in mitochondrial structure and function tend to be upregulated in T_ACT_ rather than T_ACT + PD1_ cells.

STEM also identified profile B, which included transcripts whose expression decreased with time in T_ACT_ relative to T_ACT + PD1_ cells (Fig. 4a). Profile B was enriched in GO categories related to transmembrane receptors and ion binding activities (Additional file 13: Table S5), but none of these genes was significant after multiple comparison correction.

Using qPCR in an independent set of samples, we validated the differential expression of a series of mitochondrial genes (Fig. 4c), including *HSPA9* (chaperone), *CHCHD3*, *CHCHD10* and *PHB* (cristae morphogenesis), and *MIEF1*, *MTFP1* and *MTFR2* (mitochondrial fission); repression of these genes was PD-1 dose-dependent (Additional file 14: Figure S8). Consistent with their transcriptomic upregulation, CHCHD3, CHCHD10 and MTFR2 protein levels were increased in T_ACT_ compared to T_ACT + PD1_ cells, as detected by immunoblot (Fig. 4d, e). T_ACT_ cells also showed a general tendency to upregulate other mitochondrial proteins such as the chaperone HSP60 and the fatty acid beta-oxidation protein HADHA, although variability among donors precluded significance. Expression of the mitochondrial respiratory chain proteins NDUFA9 (complex I), SDH-B (complex II), CORE II (complex III) and β-F1ATPase (complex V) showed no statistical difference between T_ACT_, T_ACT + PD1_ and T_CTRL_ cells, although NDUFA9 tended to be downregulated in T_ACT_ cells. We could not analyze expression differences in complex IV (cox-IV and cox8A) due to deficient antibody function or protein insolubility.

### PD-1 reduces mitochondrial number but does not affect dynamics

**Fig. 5:**
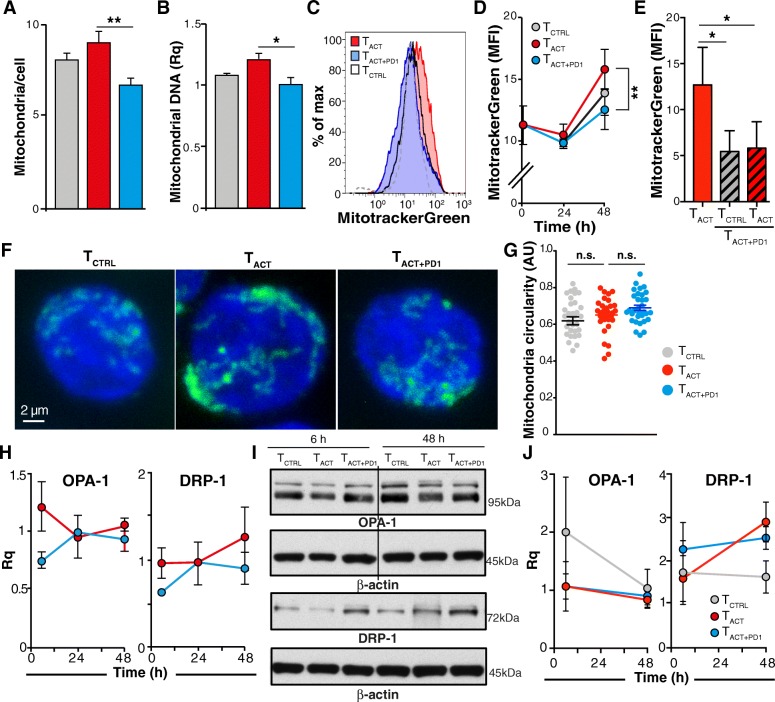
PD-1 stimulation reduces the number of mitochondria but does not affect mitochondrial dynamics. **a** Number of mitochondria per cell as determined by direct counting from transmission electron microscopy images (*n* ≥ 83 cells/condition). Results are the average of counting by two independent observers, one of them blind to the experiment. **b** Relative mitochondrial DNA quantity determined by qPCR (*n* = 3). **c** Representative histogram of T_CTRL_, T_ACT_, and T_ACT + PD1_-stimulated cells (48 h) stained with the MitoTracker Green probe. **d** Quantification of mean fluorescence intensity from cells as in **c** (*n* = 7 donors). **e** Quantification of MitoTrackerGreen mean fluorescence intensity in T_ACT_ and pre-treated T_ACT + PD1_ cells restimulated with T_CTRL_and T_ACT_ beads (*n* = 3). **f** Representative confocal images of T_CTRL_, T_ACT_, and T_ACT + PD1_ cells stained with aconitase-2. **g** Quantification of mitochondrial circularity, determined from confocal images as in **e** using ImageJ software (*n* ≥ 31 cells/condition). **h** Quantification of OPA-1 and DRP-1 mRNA levels in T_ACT_ and T_ACT + PD1_-stimulated cells. Values were normalized to those from T_CTRL_ cells. **i** Representative OPA-1 and DRP-1 immunoblots in cells treated as indicated. The line indicates removal of an empty lane. **j** Densitometric analysis of immunoblots as in **h**. The Rq was calculated as the ratio between each protein and β-actin, taking the value for T_CTRL_ cells as reference (*n* = 3 donors). In all cases, data were compared using one-way (**a**, **b**, **g**), two-way ANOVA (**d**, **h**, **j**) with Bonferroni’s post-test, or paired two-tailed Student’s *t*-test (**e**); * *p* < 0.05, ** *p* < 0.01, n.s., not significant

We analyzed whether different treatments influenced cell mitochondrial mass. HSP60 is a marker of mitochondrial biogenesis [26]. Consistent with the tendency to HSP60 downmodulation in T_ACT + PD1_ cells, mitochondrial number was significantly reduced in T_ACT + PD1_ compared to T_ACT_ cells, as determined by direct counting (Fig. 5a; Additional file 15: Figure S9A-C), relative mtDNA quantity (Fig. 5b), or MitotrackerGreen staining (Fig. 5c, d). In contrast, mitochondrial mass was statistically unchanged between T_ACT_ and T_CTRL_ cells (Fig. 5a-d). As for ΔΨm, cell pre-incubation with PD-L1 reduced mitochondrial mass, which was not reversed after their re-stimulation with T_ACT_ beads (Fig. 5e).

Mitochondrial morphology and number in T cells is influenced dynamically by the processes of fusion and fission [26]. PD-1 stimulation downregulated mRNA and protein levels of MTFR2 (Fig. 4c-e), a mitochondrial fission promoter [28]. We thus measured mitochondrial interconnectivity and shape from confocal micrographs of aconitase 2-stained T_CTRL_, T_ACT_ and T_ACT + PD1_ cells (Fig. 5f). We found no differences in mitochondrial circularity, a criterion related to fission/fusion events [29], in the cell types analyzed (Fig. 5g). Moreover, we detected no changes associated to cell treatment in mRNA or protein levels of OPA-1 or DRP-1 (Fig. 5h-j), two master regulators of mitochondrial fusion and fission [26]. Although PD-1 downmodulates MTFR2, it thus seems insufficient to substantially affect mitochondrial dynamics. In a very small number of T_ACT + PD1_ cells, we found discrete mitochondria engulfed by double-membrane structures that resembled autophagosomes (Additional file 15: Figure S9D). Nevertheless, we detected no differential expression of mitophagy-associated genes in T_ACT + PD1_ cells (not shown).

### PD-1 decreases the number and length of mitochondrial cristae

Although several reports linked PD-1 to functional mitochondrial impairment [15, 17–19], the structural changes in mitochondria from PD-1-stimulated CD8^+^ T cells have not been described in detail. PD-1 downregulated two genes, *CHCHD3* (also termed *Mic19*) and *CHCHD10* (*Mic14*; Fig. 4d, e), which form part of the mitochondrial contact site and MICOS [30]. In mammalian cells, the MICOS is a multimeric complex composed of nine known subunits and putative interactors, which links the inner boundary to the outer mitochondrial membranes and stabilizes cristae junctions [30].

**Fig. 6:**
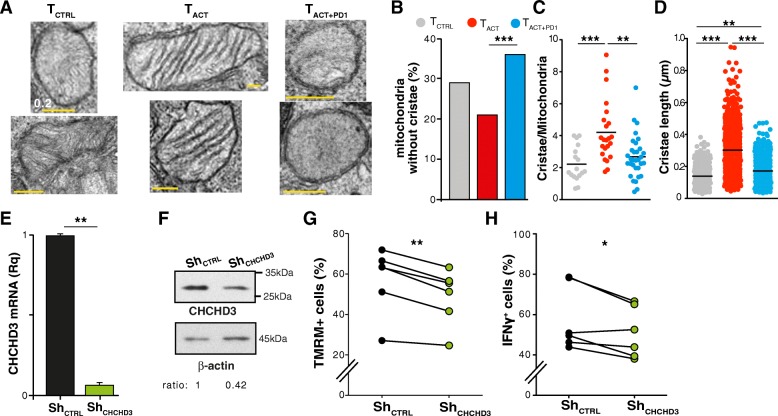
PD-1 reduces the number and length of mitochondrial cristae. **a** Representative micrographs showing magnified mitochondria from T_CTRL_, T_ACT_, and T_ACT + PD1_-stimulated cells (48 h). **b-d** Percentage of mitochondria without cristae (**b**), average number of cristae per mitochondrion in each cell (**c**), and length of cristae in each mitochondrion (**d**) in CD8^+^ T cells stimulated for 48 h, as indicated. **e** Relative CHCHD3 mRNA levels in shRNA_CTRL_- or shRNA_CHCHD3_-transduced CD8^+^ T cells. Data are mean ± SEM (*n* = 3). **f** Representative immunoblot showing CHCHD3 protein levels in shRNA_CTRL_- or shRNA_CHCHD3_-transduced cells. The densitometric CHCHD3/β-actin ratio was calculated, using the value for shRNA_CTRL_ cells as reference (*n* = 2). **g, h** Percentage of shRNA_CTRL_- or shRNA_CHCHD3_-transduced CD8^+^ T cells showing polarized mitochondria, as determined by TMRM staining (**g**), and producing IFNγ (**h**). Each pair of points represents an independent donor. For **a**, **b** and **d**, *n* = 127 (T_CTRL_), 170 (T_ACT_) and 222 (T_ACT + PD1_) mitochondria analyzed; for **c**, *n* = 17 (T_CTRL_), 23 (T_ACT_) and 33 (T_ACT + PD1_) cells. * *p* < 0.05, ** *p* < 0.01, *** *p* < 0.001, one-way ANOVA with Bonferroni’s post-test (**b**-**d**) or two-tailed paired Student’s *t*-test (**e**, **g**-**h**)

Ultrastructural analyses showed clear differences in the organization of the inner mitochondrial membrane and cristae (Fig. 6a). Mitochondria from T_ACT_ cells had a large number of tight cristae, with a parallel-oriented lamellar profile (Fig. 6a). This contrasted with the loose vesicular profile of cristae in T_CTRL_ cells. T_ACT + PD1_ cell mitochondria also had some swollen cristae, although they did not show the clear vesicular profile observed in T_CTRL_ cells (Fig. 6a); this is consistent with the loss of respiratory capacity and transcriptomic downregulation of structural proteins. Moreover, T_ACT + PD1_ cell mitochondria often lacked visible cristae (Fig. 6a). The percentage of mitochondria without cristae was significantly larger in T_ACT + PD1_ than in T_ACT_ cells (Fig. 6b). Although T_CTRL_ cells also had a larger number of mitochondria without cristae than T_ACT_ cells (Fig. 6b), the differences were not significant (*p* = 0.14; Fisher’s exact test). The number of cristae per mitochondrion and the length of these cristae was significantly reduced in T_ACT + PD1_ compared to T_ACT_ cells (Fig. 6c, d). The results suggest that PD-1-induced downmodulation of these MICOS-associated proteins affect cristae organization.

We tested whether PD-1-induced CHCHD3 downregulation is responsible for the dysfunctional state of mitochondria. Purified, activated CD8^+^ T cells were transduced with lentiviruses encoding control or CHCHD3 short hairpin (sh)RNA; transduction efficiency was 11–53%. CHCHD3-shRNA effectively downregulated CHCHD3 mRNA and protein levels at 48 h post-transduction (Fig. 6e, f). Transduced cells were then reactivated, and mitochondrial polarization and IFNγ production analyzed in shRNA-expressing cells (gated by GFP co-expression). CHCHD3 silencing caused a significant reduction in the polarization of mitochondria (Fig. 6g) and in IFNγ production (Fig. 6h), indicating that downregulation of a single MICOS-associated protein is sufficient to produce mitochondrial dysfunction and to impair T cell activation.

### Alterations in mitochondrial cristae are associated with increased supercomplex assembly

**Fig. 7:**
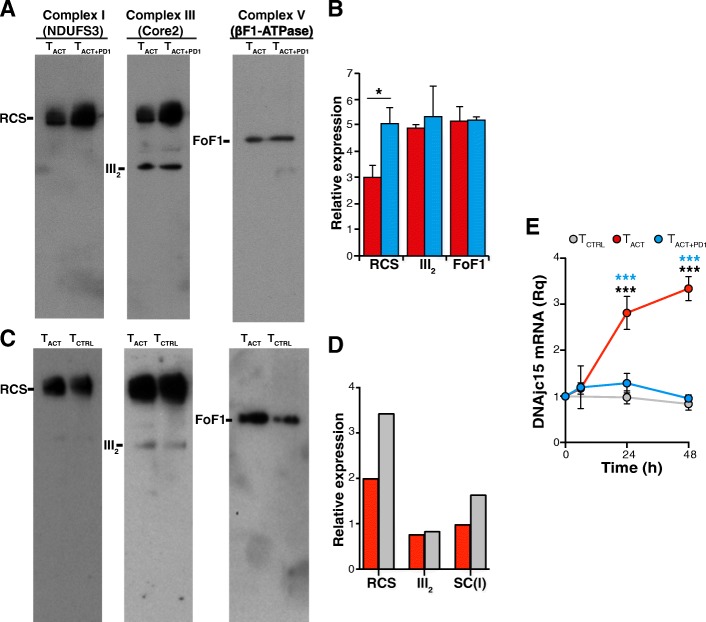
PD-1 increases the formation of supercomplexes. **a** Representative blue native PAGE showing RCS formation in T_ACT_, and T_ACT + PD1_-stimulated cells (48 h). Blots were hybridized sequentially with anti-NDUFS3 (complex I), −Core2 (complex III) and -βF1-ATPase (complex V) antibodies. **b** Densitometric quantification of the blots shown in *A* (*n* = 4; *, *p* < 0.05, paired two-tailed Student’s *t*-test). **c** Blue native PAGE showing RCS formation in T_ACT_ and T_CTRL_ cells (48 h); hybridizations were as in **a**. **d** Densitometric quantification of the blots shown in **c**. Data shown are from a pool of three donors. **e** Relative *MCJ/DnaJC15* mRNA levels in T_CTRL_, T_ACT_ and T_ACT + PD1_ cells at different times post-stimulation with indicated beads. Values were normalized to unstimulated cells (time 0). Data are mean ± SEM (*n* = 3 independent donors). *** *p* < 0.001, two-way ANOVA with Bonferroni’s post-hoc test

Individual respiratory chain complexes can be organized in quaternary supramolecular structures termed supercomplexes (RCS) [31, 32]. These RCS reside in the inner mitochondrial membrane, and establish an efficient proton gradient for complex V to synthesize ATP [33]. Although the precise RCS arrangement is largely unknown, high-resolution structural models of the mammalian respirasome have been described [34–37]. Since RCS are highly enriched in the cristae membrane [31, 32] and their formation/stability is linked to cristae shape [38], we tested whether the morphological changes in the T_ACT + PD1_ cell cristae affected RCS formation. To our surprise, we found greater enrichment of RCS containing complexes I and III in mitochondrial membranes of T_ACT + PD1_ and T_CTRL_ than of T_ACT_ cells (Fig. 7a-d); in contrast, complex III dimers were represented equally in all cell types (Fig. 7a-d).

We searched our RNA-seq data for differentially regulated genes that could explain the increased RCS formation or stability in T_ACT + PD1_ and T_CTRL_ cells, focusing on the co-chaperone MCJ (methylation-controlled J protein; also termed DnaJC15), which is described as a negative regulator of RCS formation/stability in CD8^+^ T cells [39]. We found time-dependent *MCJ/DnaJC15* mRNA upregulation in T_ACT_ compared to T_ACT + PD1_ and T_CTRL_ cells (Fig. 7e).

## Discussion

Reactivation of tumor-specific T cells through PD-1/PD-L1 axis blockade has emerged as a prominent immunotherapeutic option for many cancers. Little is known of the inhibitory signals transduced by PD-1 that hinder T cell anti-tumor activity. Several reports defined genome-wide transcriptional programs and the underlying molecular circuitry in exhausted CD8^+^ T cells, based on lymphocytes isolated from animals infected with viruses that induce exhaustion [40, 41], or from the tumor microenvironment [15, 19, 42]. Since the exhaustion program is not mediated exclusively by PD-1 signaling [43], the genetic programs identified cannot be ascribed entirely to PD-1 activity in these cells. Our system was designed to define specific genetic programs regulated after PD-1 engagement, constituting an ideal method to identify signaling pathways controlled by this inhibitory receptor. Principal component analysis and hierarchical clustering showed clear commonalities in the transcriptional programs of resting and PD-L1-stimulated cells at 6 h. In contrast, expression profiles of T_ACT + PD1_ cells stimulated for 24 and 48 h segregated from that of resting cells. Our data for human CD8^+^ T cells thus indicate that PD-1 elicits a unique, time-dependent transcriptomic program that differs from that in resting T cells. Further research is warranted to study the potential of these PD-1-induced/repressed genes in the inhibition of T cell effector function.

A set of 1651 genes showed significant divergence of expression between T_ACT_ and T_ACT + PD1_ cells, although only 578 passed the FDR correction. Signaling pathway enrichment analyses indicated metabolism as the process with the largest number of genes with differing expression between these conditions. From the metabolic pathways inferred to be targeted, we showed that PD-1 significantly reduced CD8^+^ T cell capacity to switch on glycolysis and mitochondrial respiration (determined by reduced basal and maximal OCR) following activation using glucose as a substrate. We nonetheless found that the OCR/ECAR ratio was significantly higher in T_ACT + PD1_ than in T_ACT_ cells, as reported for PD-1-stimulated CD4^+^ T cells [16, 44]. Glycolysis inhibition in PD-1-stimulated cells can be explained by the reported activation of the phosphatase PTEN and subsequent downmodulation of the AKT/mTOR pathway, downstream of PD-1 [20]. PD-1-mediated inhibition of basal and maximal respiration rates could be a result of reduced expression and/or decreased activity after covalent modification of respiratory chain proteins by phosphorylation [45–47].

Our results also suggest that metabolic changes induced by PD-1 are not simply the consequence of PD-1 inhibitory activity on T cell activation. Indeed, using palmitate as a substrate, we found that FAO-dependent OCR was higher in T_ACT + PD1_ than in resting (T_CTRL_) cells. This FAO elevation was associated to a time-dependent increase in CPT1A expression specifically in T_ACT + PD1_ cells. Moreover, relative proton leak was also lower in T_ACT + PD1_ than in T_ACT_ or T_CTRL_ cells, a phenotype reported for memory T cells, which are characterized by efficient mitochondrial respiration [48]. These data suggest that PD-1 shapes CD8^+^ T cell metabolism similar to long-lived cells, and provides a mechanistic explanation for the long-lived characteristics of tumor-infiltrating lymphocytes (TIL) in a metabolically insufficient tumor microenvironment.

Our study showed that mitochondrial number and function (impaired ΔΨm) were restrained in T_ACT + PD1_ cells. It is difficult to assess which of these two alterations is more important for explaining PD-1-induced metabolic dysfunction. It is noteworthy that the tumor microenvironment represses mitochondrial biogenesis [15], whereas 4-1BB costimulation increases mitochondria numbers in CD8^+^ T cells [49]; our data thus concur with the hypothesis that variation in mitochondria number might be a regulatory target for co-stimulatory and inhibitory receptors. RNA-seq data showed differential expression between T_ACT + PD1_ and T_ACT_ cells of 84 genes coding for mitochondrial proteins. Among these, we found mitochondria biogenesis markers such as HSP60, and some fusion/fission regulators such as MTFP1 and MTFR2, which were validated as downmodulated in PD-1-stimulated cells at both transcriptomic and protein levels. No alterations were detected between T_ACT + PD1_ and T_ACT_ cells in mitochondria circularity and interconnectivity, two criteria related to fusion/fission processes [29]. Expression of OPA-1 and DRP-1, two major regulators of mitochondria fusion/fission events, was also unaltered by PD-1 engagement. We found some images resembling mitophagy exclusively in T_ACT + PD1_ cells, although mitophagy-inducing genes were not induced in these cells. It is possible that the moderate reduction of mitochondria number in T_ACT + PD1_ cells might be a sum of discrete events.

Neither the PD-1-induced ΔΨm inhibition nor mitochondria number reduction can be rescued by PD-1 washout. These results coincide with previous reports indicating that repression of mitochondrial activity in the tumor microenvironment cannot be rescued by PD-1 blockade [15]. There is, in fact, a heritable epigenetic mechanism that drives T cell exhaustion, which is not completely reversed by anti-PD-1 blockade [50]. A mechanistic explanation for our results might thus be that PD-1 engagement caused epigenetic reprograming of CD8^+^ T cells, which led to irreversible functional alteration of the mitochondria. Further research is needed to verify this hypothesis. The irreversibility of mitochondria function as well as the preferential use of FAO in T_ACT + PD1_ cells thus suggest that PD-1 engagement induces a metabolic program different from that of resting T cells.

Ultrastructural analyses also revealed notable changes in inner mitochondrial membrane organization in T_ACT + PD1_ cells, with a severe reduction in cristae/mitochondrion length and number, or even in their complete loss. In lung cancer patients, mitochondria from TIL with high PD-1 levels show fewer and shorter cristae than those in TIL with low or no PD-1 expression [19]. The mitochondrial cristae phenotype observed here after PD-1 engagement appears to correspond to a true defect of cytotoxic lymphocytes exposed in vivo to PD-1 stimulation.

CHCHD3 is an important regulator in the organization and stability of the MICOS complex, as it links the inner and outer mitochondrial membranes through interaction with SAM50 [30]. Our analysis indicated consistent downregulation of two MICOS complex proteins, CHCHD3 and CHCHD10. Given the low transfection efficiency of primary CD8^+^ T lymphocytes, we were unsuccessful in simultaneously silencing CHCHD3 and CHCHD10. In yeast, the soluble CHCHD3 protein functions as the key component in directing the inner membrane distribution of each MICOS subcomplex [51]. We therefore postulated that CHCHD3 silencing would be sufficient to reproduce the cristae formation defects observed in PD-1-stimulated cells. CHCHD3 silencing indeed recapitulated several of the PD-1-induced dysfunctions in CD8^+^ T cells, such as the decline in mitochondrial depolarization and the reduction in IFNγ production. Given the low transduction efficiency of the siRNA, however, we were unable to assess morphological alterations in mitochondrial cristae of CHCHD3 silenced CD8^+^ T cells in our system. CHCHD3 silencing in HeLa cells nonetheless leads to notable changes in cristae morphology and even to their loss in most cells [51].

The presence of RCS has been demonstrated in many tissues and cells, including T cells [39]. These RCS place individual complexes together, which increases electron transfer efficiency in the respiratory chain and reduces ROS production. The primary function of MICOS is to stabilize, position, and control the copy number of cristae junctions to organize the inner membrane into an efficient respiratory machine [51]. Indeed, cristae remodeling by OPA1 depletion affects RCS formation and decreases respiratory efficiency [38]. We anticipated that the reduction in OCR and ΔΨm in addition to the dysmorphic cristae in T_ACT + PD1_ cells might be linked to impaired RCS formation. BN-PAGE analyses nevertheless showed that complex I- and III-containing RCS were increased in T_ACT + PD1_ compared with T_ACT_ cells. RCS were also increased in T_CTRL_ compared with T_ACT_ cells, although cristae were also defective in T_CTRL_ cells. The increased RCS assembly in T_ACT + PD1_ and T_CTRL_ cells might be a compensatory mechanism to guarantee mitochondrial respiration following severe ultrastructural disorganization of the inner membrane. The reduced complex-I-containing RCS assembly in T_ACT_ cells could be related to upregulation of the co-chaperone MCJ/DnaJC15, a negative regulator of RCS levels in cardiomyocytes and CD8^+^ T lymphocytes [39].

## Conclusions

Several studies underlined the importance of metabolic sufficiency in the initiation and maintenance of anti-tumor immunity [15, 44], and chemicals that enhance mitochondrial metabolism synergize with PD-1 blockade therapy to reduce tumor growth in mice [18]. Our studies highlight mitochondria as the main targets of PD-1 inhibitory activity, causing metabolic rewiring to FAO as well as apparently irreversible mitochondrial dysfunctions that are not simply the consequence of inhibition of the T cell activation program. We also found that structural alterations of the cristae network in PD-1-engaged or resting T cells unexpectedly triggered RCS formation. A major challenge will be to design strategies to restore the function of these newly identified elements downstream of PD-1 to reinvigorate anti-tumor immune responses in vivo.

## Additional files

Additional file 1: Supplementary Methods. Detailed description of the methods and materials used in the study. (DOCX 34 kb)

Additional file 2: **Table S1.** List of primers used for RT-qPCR analyses. (PDF 44 kb)

Additional file 3: **Figure S1.** Dose-dependent inhibition of T cell activation by PD-L1. (PDF 161 kb)

Additional file 4: **Figure S2.** MA-plots for differential expression analysis. (PDF 3199 kb)

Additional file 5: **Table S2.** Top 20 genes with the most divergent expression in the LRT model. (PDF 89 kb)

Additional file 6: **Table S3.** Pathways significantly enriched in the 578 genes selected. (PDF 56 kb)

Additional file 7: **Figure S3.** Scheme showing the metabolic pathways altered in PD-1-stimulated cells. (PDF 2970 kb)

Additional file 8: **Figure S4.** GO enrichment analysis for molecular function terms. (PDF 776 kb)

Additional file 9: **Figure S5.** GO enrichment analysis for biological processes terms. (PDF 777 kb)

Additional file 10: **Figure S6.** GO enrichment analysis for cellular components terms. (PDF 330 kb)

Additional file 11: **Figure S7.** ClueGO plot of the 84 mitochondrial genes differentially expressed after PD-1 ligation. (PDF 560 kb)

Additional file 12: **Table S4.** List of genes that partition or associate with mitochondria. (PDF 94 kb)

Additional file 13: **Table S5.** GO enrichment analysis of profile B by STEM (top 20). (PDF 84 kb)

Additional file 14: **Figure S8.** Changes in mitochondria-related gene expression is PD-L1 dose-dependent. (PDF 166 kb)

Additional file 15: **Figure S9.** Mitochondrial morphology analyzed by TEM. (PDF 5455 kb)

## Data Availability

The RNA-seq datasets generated during the current study are available in the GEO repository, accession number GSE122149. Other data and materials are available from the corresponding author upon reasonable request.

## Abbreviations

ATP: Adenosine triphosphate
DMEM: Dulbecco’s Modified Eagle Medium
DNA: Deoxyribonucleic acid
DNP: 2,4-dinitrophenol
ECAR: Extracellular acidification rate
ERK: Extracellular signal-regulated kinase
FAO: Fatty acid oxidation
FCCP: Carbonyl cyanide-4-(trifluoromethoxy) phenylhydrazone
GO: Gene ontology
HEK: Human embryonic kidney
IFNγ: Interferon-gamma
ITIM: Immunoreceptor tyrosine-based inhibition motif
ITSM: Immunoreceptor tyrosine-based switch motif;
KEGG: Kyoto encyclopedia of genes and genomes
LRT: Likelihood ratio test
MIB: Mitochondrial intermembrane space bridging
MICOS: Mitochondrial contact site and cristae organizing system
mtDNA: Mitochondrial DNA
mTOR: Mammalian target of rapamycin
OCR: Oxygen consumption rate
OXPHOS: Oxidative phosphorylation
PCR: Polymerase chain reaction
PD-1: Programmed death-1
PD-L1: Programmed death-ligand 1
PD-L2: Programmed death-ligand 2
PTEN: Phosphatase and tensin homolog
RCS: Respiratory chain supercomplexes
RNA: Ribonucleic acid
RNA-Seq: RNA sequencing
ROS: Reactive oxygen species
SHP-1: Src homology region 2 domain-containing phosphatase-1
SHP-2: Src homology region 2 domain-containing phosphatase-2
SRC: Spare respiratory capacity
STEM: Short time-series expression miner
TCR: T cell receptor
TEM: Transmission electron microscopy
TIL: Tumor-infiltrating lymphocytes
TMRM: Tetramethylrhodamine, methyl ester
