# Supplementary material for: PD-1 signaling affects cristae morphology and leads to mitochondrial dysfunction in human CD8+ T lymphocytes
Source: J Immunother Cancer. 2019 Jun 13;7:151. doi: 10.1186/s40425-019-0628-7 (PMC6567413; doi:10.1186/s40425-019-0628-7)
Supplement: Supplementary file 1 — Supplementary Methods. Detailed description of the methods and materials used in the study. (DOCX 34 kb) [file 40425_2019_628_MOESM1_ESM.docx]

**SUPPLEMENTARY METHODS**

**Cell culture**

Cells were cultured in a 5% CO_2_, humidity-saturated atmosphere at 37ºC. Human embryonic kidney (HEK)-293T cells (ATCC) were cultured in Dulbecco’s Modified Eagle Medium (DMEM; BioWest) supplemented with fetal bovine serum (10%, FBS), sodium pyruvate (1 mM) and L-glutamine (2 mM). Peripheral blood mononuclear cells (PBMC) were obtained from buffy coats from healthy donors (Centro de Transfusiones de la Comunidad de Madrid, Spain) with informed consent, using Ficoll density gradients. Human (h)CD8^+^ T cells were isolated from these PBMC by negative selection (EasySep human CD8^+^ T cell enrichment kit, Stem Cell Technologies), and cultured (1-2 x 10^6^ cells/ml) in RPMI-1640 medium (BioWest) supplemented with FBS (10%), L-glutamine (2 mM) and HEPES (10 mM). All media were supplemented with penicillin/streptomycin (100 U/mL).

**Flow cytometry**

hCD8^+^ cells were washed in PBS-staining buffer (PBS with 0.5% BSA, 1% FBS and 0.09% NaN3), F_c_ receptors were blocked with anti-CD16 and -CD32 antibodies, and incubated (4ºC, 30 min) with anti-CD25-PE (B1.49.9, Beckman-Coulter), -CD279-APC (MIH4, eBioscience), -CD69-PCy5 (TP1.553, Immunotech), and -CD8-FITC (B9.11, Beckman-Coulter) antibodies. IFNγ was detected by intracellular staining in permeabilized cells (Beckman-Coulter) pretreated with brefeldin A (10 μg/ml, 4 h, 37ºC; eBioscience) using anti-IFNγ-PE (B27, Pharmingen) antibody. Dead cells were detected with propidium iodide (2.5 μg/test, 1 min), or by LIVE/DEAD Fixable Red Dead Cell stain kit (Invitrogen) when staining involved a permeabilization step. Isotype controls were stained with mouse IgG_1_-FITC, IgG_2a_-PCy5.5, IgG_2a_-PE (Beckman Coulter), IgG1-PE (Pharmingen), and IgG1-APC (BioLegend) in the same conditions. Samples were analyzed in a Cytomics FC500 or a Gallios (Beckman Coulter) cytometer, and data analyzed using Kaluza and FlowJo software.

For mitochondrial studies, hCD8^+^ cells were resuspended in HBSS buffer and incubated (37ºC, 5% CO_2_, 20 min) with Mitotracker Green FM (MTG) (50 nM) and tetramethylrhodamine, methyl ester (TMRM) (40 nM), or MitoSOX mitochondrial superoxide indicator (3 µM; all from Thermo Fisher) where indicated. After washing, fluorescence was assessed by flow cytometry. To estimate mitochondrial membrane potential (ΔΨ*m*), the signal for 2,4-dinitrophenol (DNP; 750 μM)-treated cells was subtracted from that for untreated cells. Dead cells were excluded based on diamino-2-phenylindol (DAPI) staining (3 μM, 4º C, 10 min).

**Cell proliferation assay**

hCD8^+^ T cells (1 x 10^5^) cultured in 96-well plates were incubated (37ºC, 16 h) with [methyl-^3^H] thymidine (1 µCi/well; Perkin Elmer). Cells were harvested (Filtermate Harvester, Perkin Elmer) and ^3^H-TdR incorporated into DNA determined in a 1450 Microbeta liquid scintillation counter (Perkin Elmer). All samples were analyzed in quadruplicate.

**RNA sample preparation and sequencing**

The RNASeq libraries were prepared using an Illumina TruSeq Stranded Total RNA Sample Preparation kit (Illumina). Briefly, rRNA was depleted from 0.5 µg total RNA using the RiboZero Magnetic Gold Kit and fragmented by divalent cations at high temperature, resulting in 80-450-nt fragments, with the major peak at 160 nt. First and second strand synthesis was performed, the latter in the presence of dUTP instead of dTTP, to achieve directionality. The Illumina-barcoded adapters were used for adapter ligation. Libraries were enriched with PCR 15 cycles. Library size and quality was assessed in an Agilent DNA 7500 Bioanalyzer assay (Agilent).

Each library was sequenced using TruSeq SBS Kit v3-HS, in paired end mode with read length 2x 76bp. We generated on average 36 million paired-end reads for each sample in a fraction of a sequencing lane on HiSeq2000 (Illumina), following the manufacturer’s protocol. Images analysis, base calling, and quality scoring of the run were processed using the manufacturer’s Real Time Analysis (RTA 1.13.48) software, followed by generation of FASTQ sequence files by CASAVA 1.8.

**RNA-seq data processing and analysis**

RNA-seq reads were aligned against the human reference genome (gencode v19) with the GEMtools RNA-seq pipeline v1.7 ([http://gemtools.github.io](http://gemtools.github.io/)), which is based on the GEM mapper [1]. Expression quantification at the gene level was calculated with Flux (http://sammeth.net/confluence/display/FLUX/Home). RNA-seq data were analyzed using the DESeq2 R Bioconductor package, which uses a negative binomial distribution model to test for differential expression [2]. Raw counts of sequencing reads were normalized to effective library size. Gene expression levels of T_ACT_ and T_ACT+PD1_ cells at each time point were compared with that of T_CTRL_ cells, considered as time 0 for each experiment.

A likelihood ratio test (LRT) was performed using a design formula that models the class (TACT, TACT+PD1) difference over time by including an interaction term (class:time). The test is expected to find class-specific differences over time. Genes with significant p values from this test are those that at one or more time points after time 0 show a class-specific effect, which is measured as a p value for interaction (pinter) and FC values for TACT+PD1 vs. TACT cells at each time point. Genes selected by the LRT model were analyzed for enrichment in KEGG signaling pathways using the online tool Webgestalt (<http://bioinfo.vanderbilt.edu/webgestalt/>). Genes involved in metabolic pathways (KEGG hsa011000) were further explored for known interactions using Cytoescape (<http://www.cytoscape.org/>). Gene Ontology database (<http://geneontology.org/>) enrichment analysis [3]was performed using BiNGO; GO categories were summarized and visualized using ClueFO or REVIGO. Genes with a significant pinter were analyzed using STEM software for cluster analysis and integration with GO.

**Real-time quantitative PCR (RT-qPCR)**

Total RNA was extracted from hCD8^+^ cells with the RNeasy Micro Kit (up to 5 x 10^5^) or the RNeasy Mini Kit (up to 1 x 10^7^; Qiagen), and retrotranscribed (0.2-2 µg) with the High Capacity Reverse transcription Kit (Life Technologies) using random primers. mRNA levels for specific genes were quantified by RT-qPCR using indicated primers (Suppl. Table S1) and a Hot FIREPol EvaGreen Plus-based system (Solis BioDyne) in an ABI PRISM7900HT system (Applied Biosystems). After normalization to 18S ribosomal mRNA, the relative quantity (Rq) for each mRNA was calculated using the 2^−ΔΔCt^ method, taking the sample with the lowest Ct value as reference.

**Metabolic assays**

Cellular oxygen consumption (OCR) and extracellular acidification rates (ECAR) were determined in an XF24 Extracellular Flux Analyzer (SeaHorse Bioscience; Agilent Technologies) using the XF cell Mito Stress Kit (SeaHorse Bioscience). Briefly, 7 x 10^5^ T_CTRL_, T_ACT_ and T_ACT+PD1_ cells were seeded in poly-L-lysine (50 μg/ml)-coated XF24-well microplates, in non-buffered basal medium (pH 7.4, Seahorse XF Base Medium) supplemented with 25 mM glucose (Sigma-Aldrich), 2 mM L-glutamine and 1 mM sodium pyruvate (both from BioWest). Plates were centrifuged (200×*g*, 1 min) and incubated (37ºC, 30 min) to allow cell adhesion. Medium (600 μl) was then added and after 30 min, OCR and ECAR measurements were made in basal conditions and after sequential addition of oligomycin (1 μM), FCCP (1.5 μM) and rotenone/antimycin A (0.75 μM each).

FAO was determined in Krebs-Henseleit buffer (KHB) supplemented with 0.5 mM carnitine (Sigma-Aldrich), 2.5 mM glucose and 5 mM Hepes using the Agilent Seahorse XF96 Extracelular Flux Analyzer. T_CTRL_, T_ACT_ and T_ACT+PD1_ stimulated cells (48 h) were seeded (4x10^5^/well) onto Cell-Tak (1 μg/well)-coated XF96 plates, centrifuged (200×*g*, 10 sec) and allowed to attach (45 min, 37º C). Some wells were treated with etomoxir (40 μM, 15 min; Sigma-Aldrich) prior to the addition of the palmitate-BSA (167 μM; SeaHorse Bioscience). OCR was determined in basal state and after sequential injections of oligomycin (1.5 μM), FCCP (1.5 μM) and rotenone/antimycin A (1μM; all from SeaHorse Bioscience).

Lactate levels were determined enzymatically in extracts from T_CTRL_, T_ACT_ and T_ACT+PD1_ cells after 48 h stimulation, using a fluorometric lactate assay kit (Cell Biolabs) according to the supplier’s protocol. Briefly, 6 x 10^6^ cells were lysed in Assay Buffer by passage through a 30g needle. Soluble material obtained after centrifugation (12,000x*g*, 4ºC, 30 min) was assayed in black 96-well plates and fluorescence quantified in a Filter Max F5 microplate reader (Molecular Devices) at 530/590 nm excitation/emission. A lactate standard curve was run in all assays and used to extrapolate relative fluorescent units (RFU) measured in the samples. Values were obtained from duplicates and normalized to cell number.

**Blue native and immunoblot analyses**

Cells were lysed (50 mM Tris-HCl pH 8.0, 20 mM EDTA, 0.5% Triton X-100) in the presence of protease (1 mM PMSF, 10 μg/ml leupeptin, 10 μg/ml aprotinin) and phosphatase inhibitors (5 mM NaF, 1 mM Na_3_VO_4_). To detect CHCHD3, cells were lysed with RIPA buffer plus inhibitors. Protein extracts were quantified with the Micro BCA Protein Assay kit (Pierce) before SDS-PAGE analysis. Proteins were transferred to nitrocellulose membranes and immunoblotted with anti-CHCHD3 (HPA042935), -CHCHD10 (HPA003440), -MTFR2, (HPA029792), -CPT1a (HPA008835) or -β-actin (AC-15) antibodies from Sigma-Aldrich, anti-Core2 (Ab14745), -MFN1 (Ab57602), -NDUSF3 (Ab110246), -NDUFA9 (Ab14713) or -HDHA (Ab54477) antibodies from Abcam, anti-SDH-B (459230; Thermo Fisher), anti-DRP-1 (611112) or -OPA-1 (612606) from BD Biosciences, and anti-HSP60 and ‑β‑F1ATPase, as described [4]. Filters were stripped by incubation (56ºC, 30 min) in 65.5 mM Tris-HCl, 2% SDS and 100 mM 2-mercaptoethanol buffer.

For blue native analyses, we obtained a mitochondria-enriched fraction by cell lysis with a hypotonic buffer (83 mM sucrose, 10 mM MOPS pH 7.2; 30 min) and homogenization with a polypropylene pestle homogenizer and diluted in an hypertonic buffer (250 mM glucose, 30 mM MOPS pH7.2). Nuclei and unbroken cells were removed by centrifugation (1000×*g,* 10 min). Mitochondria were obtained by centrifugation (12000×*g*) from the cytosolic fraction and washed in buffer A (320 mM sucrose, 1 mM EDTA, 10 mM Tris–HCl pH 7.4). All steps were performed at 4ºC. The enriched mitochondrial fraction was suspended in 50 mM Tris-HCl pH 7.0, containing 1 M 6-aminohexanoic acid. Membranes were solubilized by adding 10% digitonin at 4g/g of mitochondrial proteins. Serva Blue G dye (5%) in 1 M 6‑aminohexanoic acid was added and mitochondrial proteins fractionated in blue native gels. Gels were electroblotted onto PVDF membranes and immunoblotted with indicated antibodies.

**Determination of mitochondria parameters by immunofluorescence**

hCD8^+^ cells were plated (37ºC, 5% CO_2_, 1 h) onto poly-L-lysine (50 µg/ml)-coated glass slides (8 wells, EM Sciences), fixed in PFA (4%, RT, 10 min), permeabilized with Triton X‑100 (0.1%, RT, 5 min) and blocked with PBS-staining buffer. Cells were then stained sequentially with anti-human aconitase-2 (4ºC, 14 h; 6F12BD9, Abcam) and goat anti-mouse Alexa 488 (RT, 1 h; Molecular Probes). Samples were mounted in Prolong Gold Antifade Reagent with DAPI (Cell Signaling). Images were captured in a Leica Microsystems microscope (LAS X v2.01) with a 60x objective. Mitochondrial morphology was determined with the ImageJ mitochondrial macro [5].

**Mitochondria ultrastructural studies by transmission electron microscopy**

After 48 h stimulation, T_CTRL_, T_ACT_ and T_ACT+PD1_ cells were washed with PBS, centrifuged and fixed (0.4 M HEPES pH 7.2, 2% glutaraldehyde, 1% tannic acid; RT, 2 h). After washing (0.4 M HEPES), samples were treated sequentially with 1% osmium tetroxide in PBS (TAAB Laboratories; 4ºC, 1 h) and 2% aqueous uranyl acetate (4ºC, 40 min). After washing with distilled water, samples were dehydrated with increasing concentrations of acetone (4ºC) and embedded in EPON 812 resin and polymerized (60ºC, 48 h). Ultrathin sections (70 nm-thick) were obtained with the Ultracut EM UC6 (Leica Microsystems), transferred to 200 mesh nickel EM grids (Gilder), and stained with 3% aqueous uranyl acetate (20 min) and lead citrate (2 min). Samples were analyzed on a JEOL JEM 1011 electron microscope (operating at 100 kV) and micrographs captured with a Gatan Erlangshen ES 1000 W digital camera at different magnifications. The number of mitochondria per cell and cristae length were quantified by two independent observers blind to the experiment.

**CHCHD3 silencing experiments**

HEK-293T cells were co-transfected with the third-generation transfection system and lentiviral plasmids coding for CHCHD3 or control (HSH013866-LVRU6GP) short hairpin RNA (shRNA; Genecopoeia). Viral supernatant was collected at 48 h post-transfection and concentrated by centrifugation (18000x*g*, 4ºC, 2 h,). Prior to transduction, hCD8^+^ cells were stimulated (24 h) with anti-CD3 and -CD28 antibody-coated beads. Activated cells were transduced with viral supernatants (titer 1-2 x 10^8^ TU/ml) at 10-20 m.o.i in the presence of polybrene (8 μg/ml, Sigma) by spinoculation (800x*g*, 2 h, 37ºC). CHCHD3 silencing was determined by qPCR and immunoblot at 48 h post-infection. TMRM staining, IFNγ production, and CD25/CD69 levels were determined by flow cytometry in EGFP^+^ gated cells, as above.

1. Marco-Sola S, Sammeth M, Guigo R, Ribeca P. The GEM mapper: fast, accurate and versatile alignment by filtration. Nat Methods. 2012; 9(12):1185-8.

2. Love MI, Huber W, Anders S. Moderated estimation of fold change and dispersion for RNA-seq data with DESeq2. Genome Biol. 2014; 15(12):550.

3. Ernst J, Bar-Joseph Z. STEM: a tool for the analysis of short time series gene expression data. BMC Bioinformatics. 2006; 7:191.

4. Acebo P, Giner D, Calvo P, Blanco-Rivero A, Ortega AD, Fernandez PL *et al*. Cancer abolishes the tissue type-specific differences in the phenotype of energetic metabolism. Transl Oncol. 2009; 2(3):138-45.

5. Dagda RK, Cherra SJ, 3rd, Kulich SM, Tandon A, Park D, Chu CT. Loss of PINK1 function promotes mitophagy through effects on oxidative stress and mitochondrial fission. J Biol Chem. 2009; 284(20):13843-55.
