# Supplementary material for: PD-1 signaling affects cristae morphology and leads to mitochondrial dysfunction in human CD8+ T lymphocytes
Source: J Immunother Cancer. 2019 Jun 13;7:151. doi: 10.1186/s40425-019-0628-7 (PMC6567413; doi:10.1186/s40425-019-0628-7)
Supplement: Supplementary file 2 — Table S1. List of primers used for RT-qPCR analyses. (PDF 44 kb) [file 40425_2019_628_MOESM2_ESM.pdf]

Table S1. List of primers used for RT-qPCR analyses

Gene Symbol	Primer (5' – 3') Forward	Reverse
CHCHD3	AGGCGGACGAGAATGAGAAC	GCACCAGAATACCGCTGAGA
CHCHD10	CTGTGGGACACGTCATGG	AACTGCCTGATCTCGTAGGC
DNAJC15	TCGACCAGCAGAGACTGGTA	AGATCCGAAATGCGTAGCGA
DRP-1	AGAAAATGGGGTGGAAGCAGA	TGAATTGGTTCAGGGCTTACTCC
HSPA9	GGACTATCGCTCCATGCCAA	TGGGGCTCTGCCAAAAAGAT
MIEF	GGGACATGTACTTGAGTGGCA	CAAGGGGCACAATGAGTTGG
MTFP1	CTTTCCGCTCTCTTGTGCCA	TCCAGGAGGAAATCCACCGA
MTFR2	CTTCGCTCTCAGATTGCAGC	ACCCAAACTAATGCGCTCGT
OPA-1	GTGGCGACTACGTCGGG	AATGCTTCGTGAAACCAGATG
PHB	ATCCCGTGGGTACAGAAACC	GAGGATGCGCAGTGTGATGT
18S rRNA	GAGAAACGGCTACCACATCC	GGGTCGGGAGTGGGTAAT
