# Supplementary material for: PD-1 signaling affects cristae morphology and leads to mitochondrial dysfunction in human CD8+ T lymphocytes
Source: J Immunother Cancer. 2019 Jun 13;7:151. doi: 10.1186/s40425-019-0628-7 (PMC6567413; doi:10.1186/s40425-019-0628-7)
Supplement: Supplementary file 3 — Figure S1. Dose-dependent inhibition of T cell activation by PD-L1. (PDF 161 kb) [file 40425_2019_628_MOESM3_ESM.pdf]

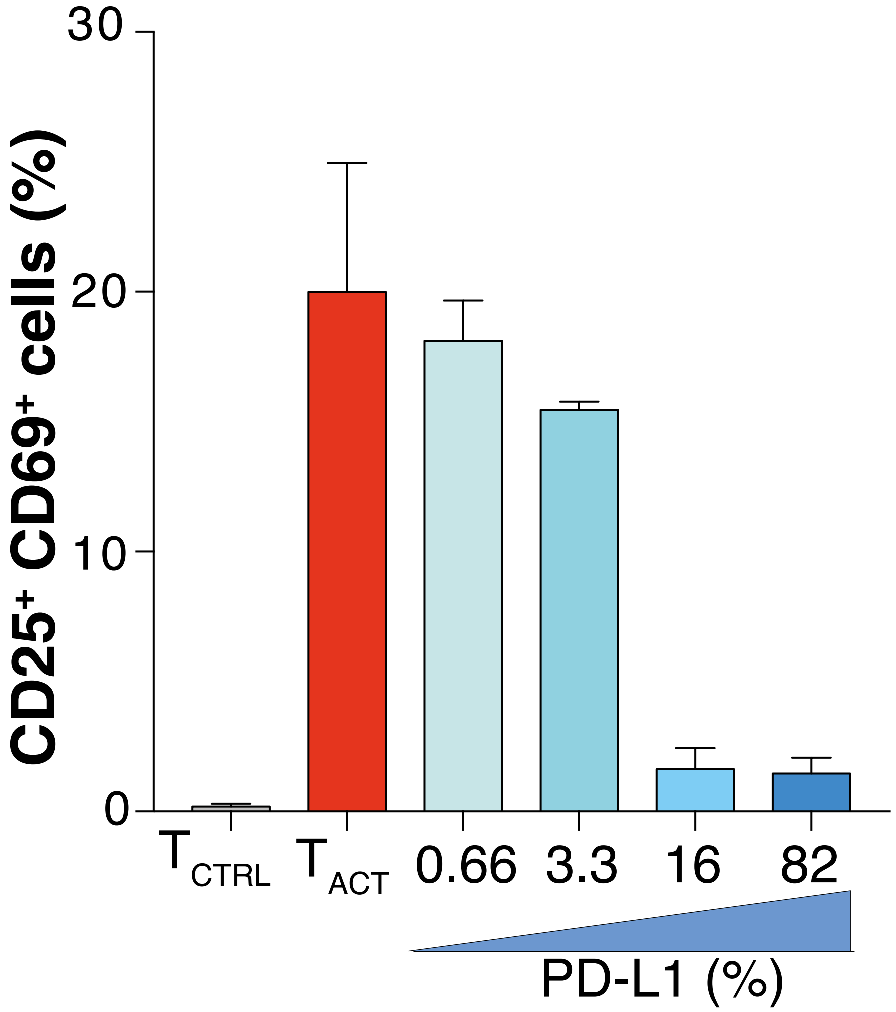

Figure S1. Dose-dependent inhibition of T cell activation by PD-L1. Primary human $CD8^+$ T cells were incubated (48 h) with beads coated with IgG (T_{CTRL}), anti-CD3 (8%) + anti-CD28 (10%) activating antibodies (T_{ACT}), or with anti-CD3+anti-CD28 plus PD-L1-Fc at 82, 16.4, 3.3 or 0.66%. Cells were stained with anti-CD25 and -CD69 antibodies and analyzed by FACS. Quantification of CD25- and CD69-expressing cells from two donors in each condition.
