# Supplementary material for: PD-1 signaling affects cristae morphology and leads to mitochondrial dysfunction in human CD8+ T lymphocytes
Source: J Immunother Cancer. 2019 Jun 13;7:151. doi: 10.1186/s40425-019-0628-7 (PMC6567413; doi:10.1186/s40425-019-0628-7)
Supplement: Supplementary file 4 — Figure S2. MA-plots for differential expression analysis. (PDF 3199 kb) [file 40425_2019_628_MOESM4_ESM.pdf]

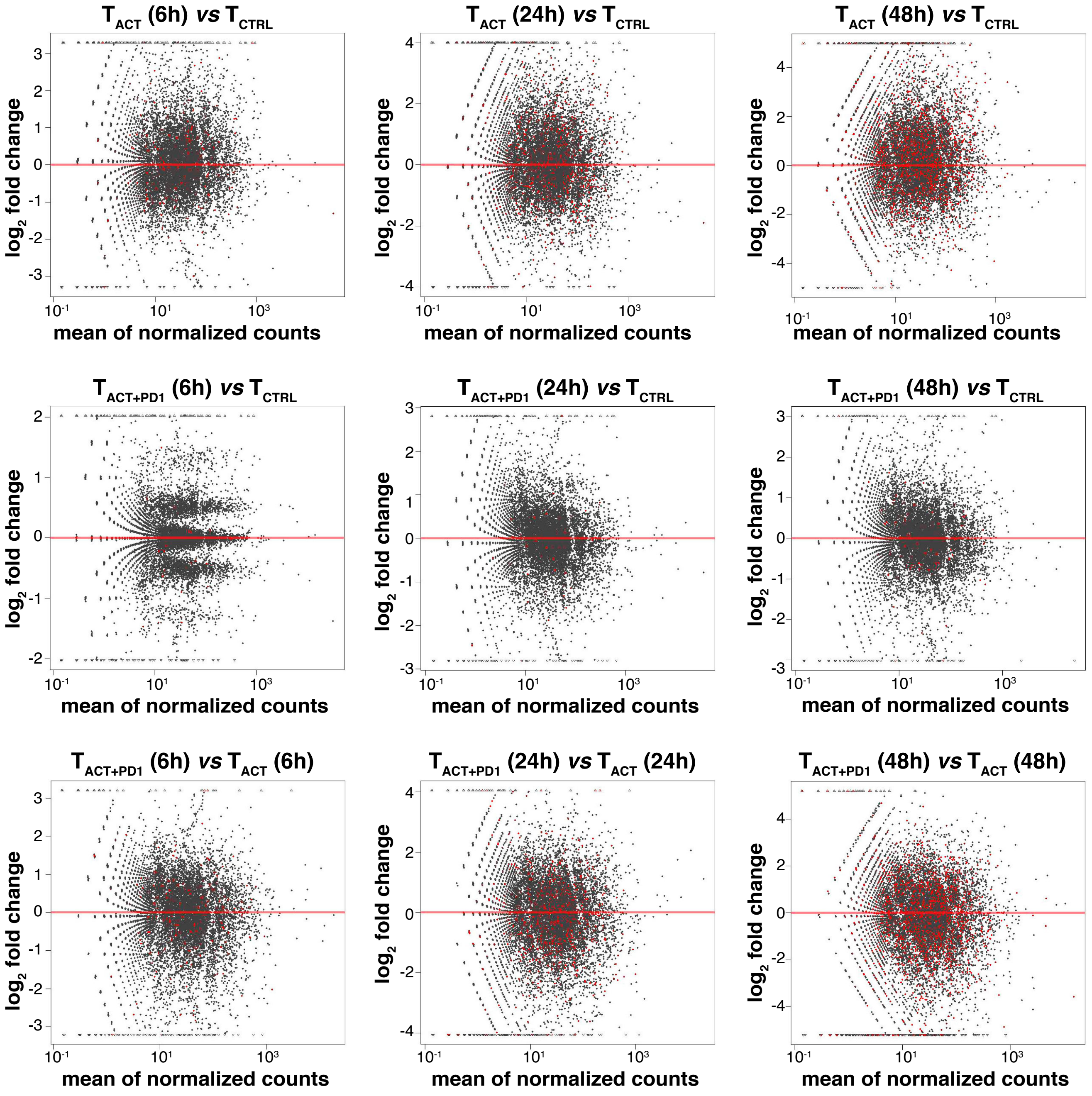

Figure S2. Differential expression analysis. MA-plots of normalized mean expression versus log2 fold change for the indicated sample pairs, as inferred from RNA-seq data. Red dots indicate differentially expressed genes statistically significant at FDR ≤ 10%.
