# Supplementary material for: PD-1 signaling affects cristae morphology and leads to mitochondrial dysfunction in human CD8+ T lymphocytes
Source: J Immunother Cancer. 2019 Jun 13;7:151. doi: 10.1186/s40425-019-0628-7 (PMC6567413; doi:10.1186/s40425-019-0628-7)
Supplement: Supplementary file 5 — Table S2. Top 20 genes with the most divergent expression in the LRT model. (PDF 89 kb) [file 40425_2019_628_MOESM5_ESM.pdf]

Table S2. Top 20 genes with the most divergent expression in the LRT model

Gene Symbol	T_{ACT} *vs* T_{CTRL}			$T_{ACT+PDL1}$ *vs* T_{CTRL}			$T_{ACT+PDL1}$ *vs* T_{ACT}			p-val (inter)	Adj. p-val (inter)
	6h	24h	48h	6h	24h	48h	6h	24h	48h		
IRF4	5.11	4.80	4.03	0.57	-0.48	-0.63	-4.55	-5.28	-4.66	7.2x10^{-22}	8.0x10^{-18}
KLF2	-1.31	-3.78	-4.70	-0.07	0.19	0.40	1.23	3.97	5.10	6.7x10^{-18}	3.7x10^{-14}
HCLS1	-0.05	-0.23	-0.33	-3.22	-0.22	-0.17	-3.17	0.01	0.16	7.5x10^{-17}	2.1x10^{-13}
TRAPPC8	-3.10	-3.41	-0.65	0.02	-0.11	-0.11	3.12	3.30	0.54	6.3x10^{-17}	2.1x10^{-13}
MKI67	-0.61	0.55	6.35	-0.27	-0.02	-0.09	0.35	-0.57	-6.45	3.3x10^{-16}	5.2x10^{-13}
NUSAP1	-0.62	1.00	5.02	-0.27	-0.38	-0.69	0.35	-1.37	-5.70	3.0x10^{-16}	5.2x10^{-13}
SEPT11	1.18	1.79	2.37	0.38	0.89	-2.34	-0.80	-0.89	-4.72	3.0x10^{-16}	5.2x10^{-13}
KLC1	-0.18	-3.30	-0.66	-0.01	0.00	0.08	0.16	3.30	0.74	8.1x10^{-14}	1.1x10^{-10}
HIST1H2AM	-0.05	0.88	4.67	0.06	0.35	0.31	0.11	-0.53	-4.36	3.1x10^{-13}	3.8x10^{-10}
CASC5	-0.39	1.74	5.10	-0.50	-0.91	-0.74	-0.11	-2.66	-5.84	1.0x10^{-12}	1.1x10^{-9}
ASPM	-0.40	1.16	6.49	-0.23	-0.10	-0.32	0.16	-1.26	-6.81	1.3x10^{-12}	1.3x10^{-9}
TOP2A	0.35	1.94	7.19	0.08	-0.83	-0.42	-0.27	-2.78	-7.61	2.4x10^{-12}	2.2x10^{-9}
PRC1	-0.65	0.04	4.15	0.06	0.09	-0.42	0.71	0.05	-4.57	3.0x10^{-12}	2.5x10^{-9}
RAB3GAP2	-2.72	-0.06	-0.02	-0.02	-0.12	-0.14	2.69	-0.06	-0.11	6.0x10^{-12}	4.8x10^{-9}
ORC1	-0.56	3.41	5.07	-0.14	-0.76	-0.75	0.42	-4.17	-5.82	9.2x10^{-12}	6.8x10^{-9}
VDR	1.40	3.96	4.07	-0.12	-1.35	-1.91	-1.52	-5.31	-5.98	1.4x10^{-11}	9.8x10^{-9}
RBBP8	1.71	2.34	3.85	-0.69	-0.97	-1.40	-2.40	-3.31	-5.25	2.6x10^{-11}	1.7x10^{-8}
HIST1H3B	0.09	2.29	7.79	0.40	-0.70	-1.68	0.31	-2.99	-9.47	3.9x10^{-11}	2.4x10^{-8}
CTB-133G6.1	-2.10	-3.25	-4.04	-0.05	0.36	0.49	2.05	3.61	4.53	5.1x10^{-11}	2.9x10^{-8}
HIST1H2AH	-0.91	2.13	6.31	0.00	0.31	0.63	0.91	-1.83	-5.68	1.0x10^{-10}	5.6x10^{-8}

Values in the comparisons are expressed as logarithm of the fold-change (FC). Adj. p-val, adjusted p-value by the multiple testing method.
