# Supplementary material for: PD-1 signaling affects cristae morphology and leads to mitochondrial dysfunction in human CD8+ T lymphocytes
Source: J Immunother Cancer. 2019 Jun 13;7:151. doi: 10.1186/s40425-019-0628-7 (PMC6567413; doi:10.1186/s40425-019-0628-7)
Supplement: Supplementary file 6 — Table S3. Pathways significantly enriched in the 578 genes selected. (PDF 56 kb) [file 40425_2019_628_MOESM6_ESM.pdf]

Table S3. Pathways significantly enriched in the 578 genes selected

Pathway name	Genes (Entrez Id)	Statistics
Systemic lupus erythematosus	126961 8367 8359 8349 85235 8358 8350 3115 3177728360 8356 440689 8365 8329 8364 8345 8346 83328335 8331 8348 8352 8968 3458 3018 8336 8341 83408357 8344 8361 3012 8334 8342 8351 3014 8337 8343	rP = 1.75 x 10^{-39}<br>aP = 2.01 x 10^{-37}
Cell cycle	9134 5347 1019 9232 6502 1111 990 4998 7531 8914176 983 4173 1031 7465 890 5111 1647 8317 41744085 1869 699 894 7533 1017 9700 23594	rP = 1.46 x 10^{-26}<br>aP = 8.39 x 10^{-25}
DNA replication	4173 5984 5111 5558 5422 4174 1763 10535 4176 59822237	rP = 7.77 x 10^{-13}<br>aP = 2.98 x 10^{-11}
Metabolic pathways	6723 10007 8681 84890 7371 586 4706 522 4709 2804245 441531 1854 7385 6472 1312 2023 8398 49074522 35 516 2954 2597 29968 5558 5333 5422 624125902 3615 952 5226 10797 4830 3703 5634 135155790 2821 10380 4713 8992	rP = 6.28 x 10^{-10}<br>aP = 1.81 x 10^{-8}
Oocyte meiosis	9134 5347 4085 9232 109 6790 699 7531 891 75331017 9700 983	rP = 2.98 x 10^{-9}<br>aP = 6.85 x 10^{-8}
p53 signaling pathway	9134 1647 1019 6241 1111 891 894 1017 983 3486	rP = 1.99 x 10^{-8}<br>aP = 3.81 x 10^{-7}
Cytokine-cytokine receptor interaction	3458 3595 3604 10913 6375 1435 9560 6351 8784 35703559 2323 7292 5008	rP = 1.25 x 10^{-5}<br>aP = 0.0002
Progesterone-mediated oocyte maturation	5347 109 699 890 891 1017 983 4085	rP = 1.77 x 10^{-5}<br>aP = 0.0003
One carbon pool by folate	6472 10797 25902 4522	rP = 7.66 x 10^{-5}<br>aP = 0.001
Huntington's disease	516 3064 1351 7385 4706 7416 522 4713 6667 4709	rP = 0.0002<br>aP = 0.0023

rP, p-value from hypergeometric tests; aP, adjusted p-value by the multiple testing method.
