# Supplementary material for: PD-1 signaling affects cristae morphology and leads to mitochondrial dysfunction in human CD8+ T lymphocytes
Source: J Immunother Cancer. 2019 Jun 13;7:151. doi: 10.1186/s40425-019-0628-7 (PMC6567413; doi:10.1186/s40425-019-0628-7)
Supplement: Supplementary file 7 — Figure S3. Scheme showing the metabolic pathways altered in PD-1-stimulated cells. (PDF 2970 kb) [file 40425_2019_628_MOESM7_ESM.pdf]

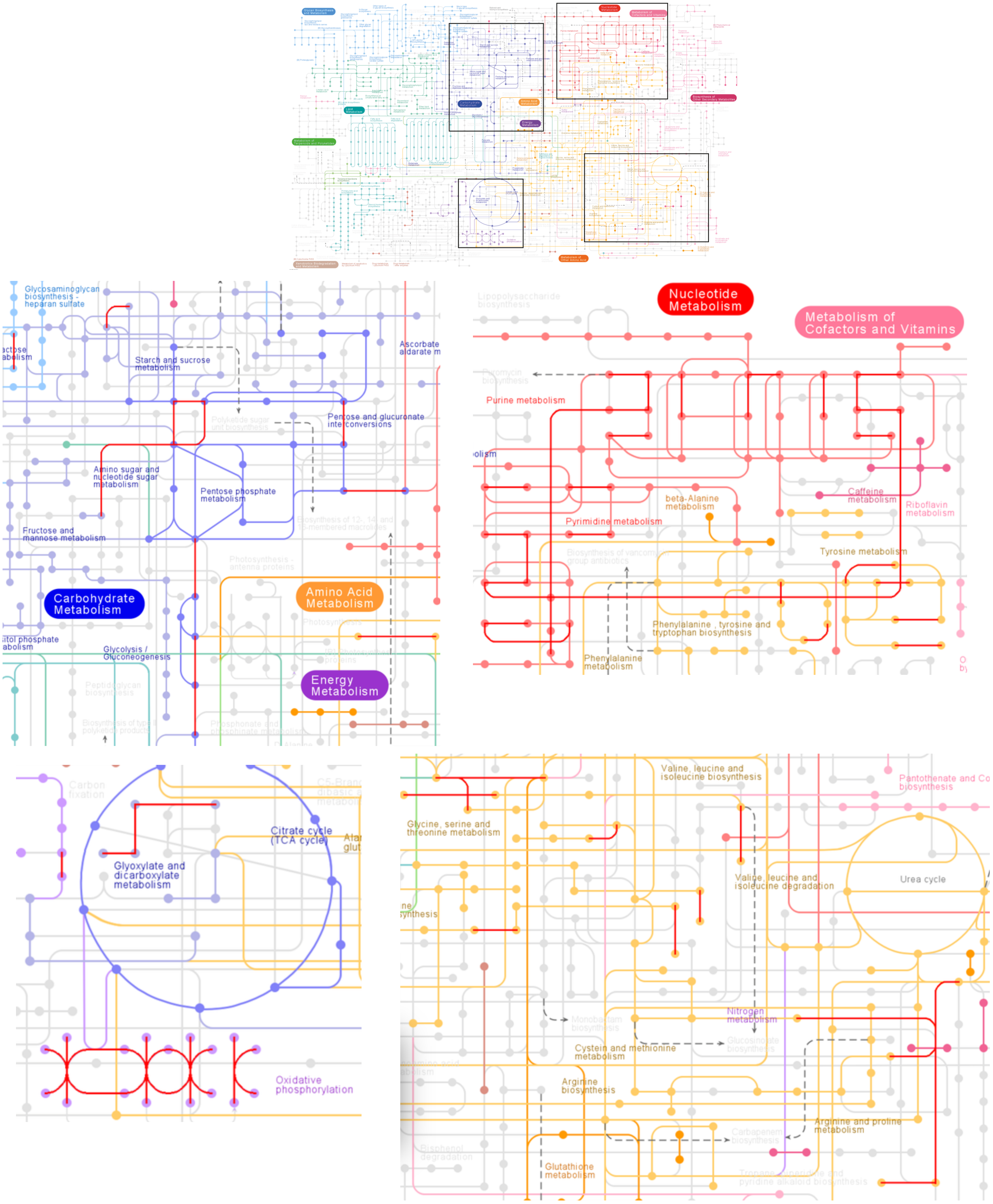

Figure S3. Metabolic pathways altered in PD-1-stimulated cells. General view of the KEGG hsa01100 “Metabolic pathways” scheme (top graph), and magnification of some metabolic routes with genes differentially expressed between T_{ACT} and $T_{ACT+PD1}$ cells (red lines).
