# Supplementary material for: PD-1 signaling affects cristae morphology and leads to mitochondrial dysfunction in human CD8+ T lymphocytes
Source: J Immunother Cancer. 2019 Jun 13;7:151. doi: 10.1186/s40425-019-0628-7 (PMC6567413; doi:10.1186/s40425-019-0628-7)
Supplement: Supplementary file 8 — Figure S4. GO enrichment analysis for molecular function terms. (PDF 776 kb) [file 40425_2019_628_MOESM8_ESM.pdf]

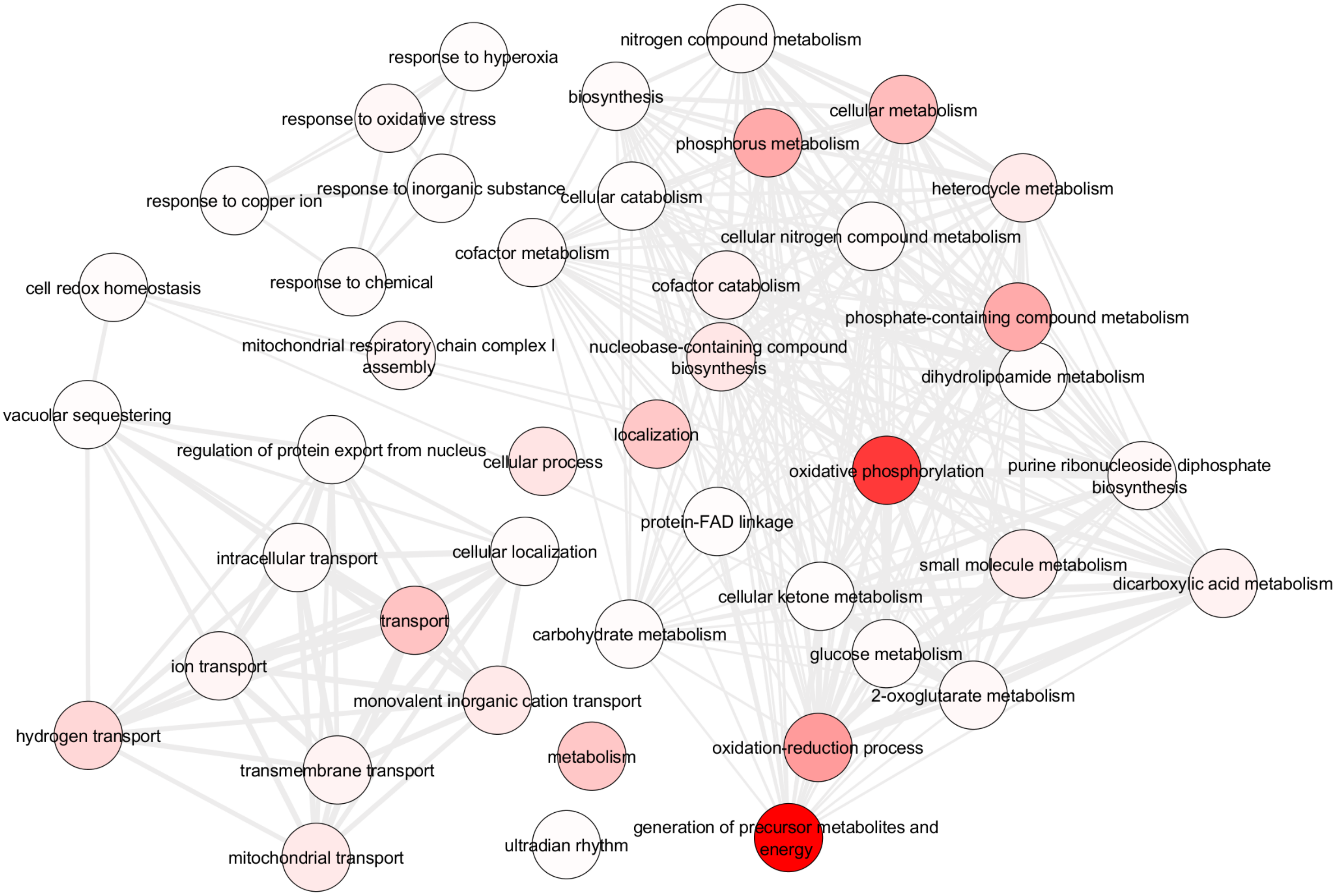

Figure S4. GO enrichment analysis for molecular function terms. REVIGO-generated network depicting relationships and overlap among significantly enriched terms in the molecular function category. Enrichment score is indicated by color (darker red indicates greater enrichment).
