# Supplementary material for: PD-1 signaling affects cristae morphology and leads to mitochondrial dysfunction in human CD8+ T lymphocytes
Source: J Immunother Cancer. 2019 Jun 13;7:151. doi: 10.1186/s40425-019-0628-7 (PMC6567413; doi:10.1186/s40425-019-0628-7)
Supplement: Supplementary file 10 — Figure S6. GO enrichment analysis for cellular components terms. (PDF 330 kb) [file 40425_2019_628_MOESM10_ESM.pdf]

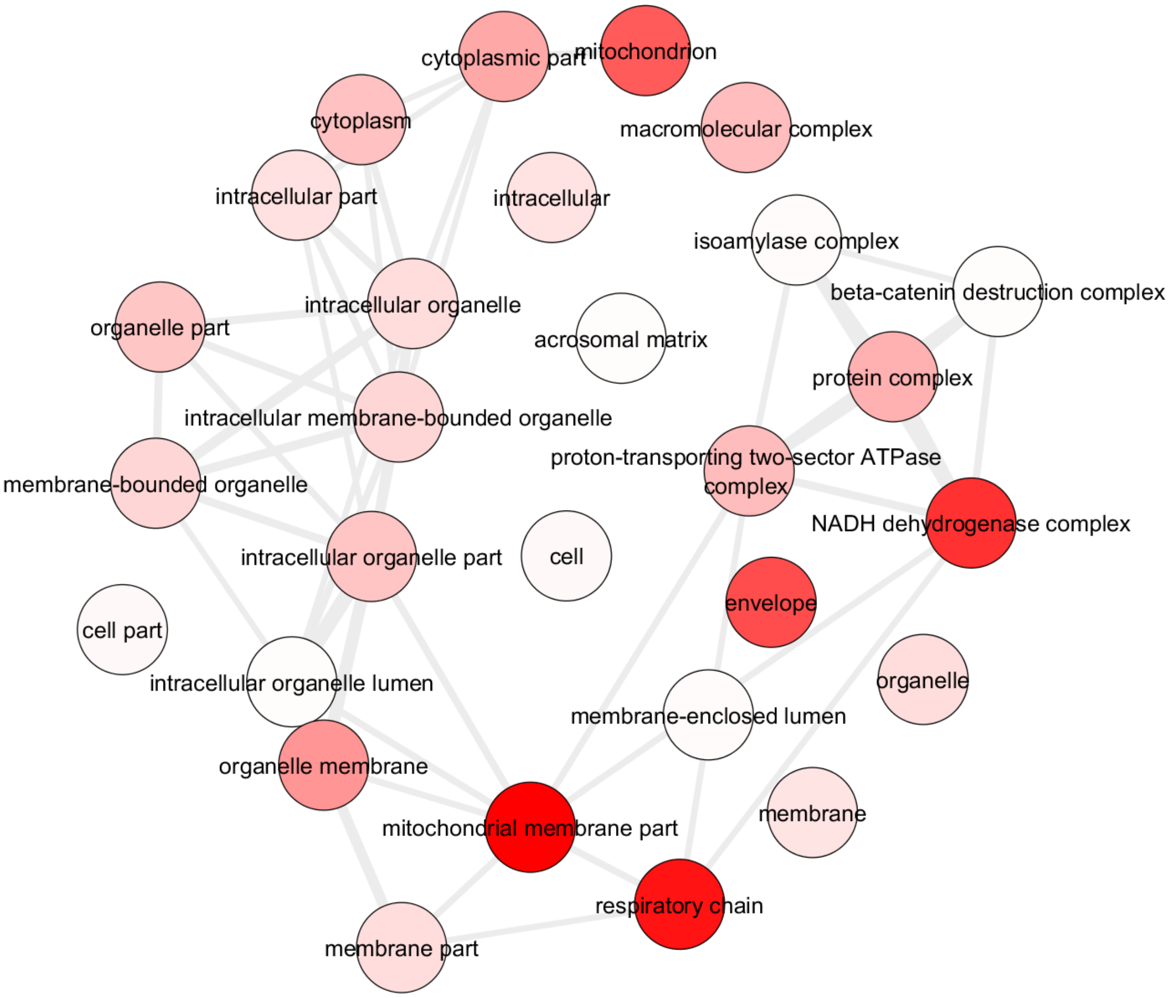

Figure S6. GO enrichment analysis for cellular components. REVIGO-generated network depicting relationships and overlap among significantly enriched cellular components. Enrichment score is indicated by color (darker red indicates greater enrichment).
