# Supplementary material for: PD-1 signaling affects cristae morphology and leads to mitochondrial dysfunction in human CD8+ T lymphocytes
Source: J Immunother Cancer. 2019 Jun 13;7:151. doi: 10.1186/s40425-019-0628-7 (PMC6567413; doi:10.1186/s40425-019-0628-7)
Supplement: Supplementary file 11 — Figure S7. ClueGO plot of the 84 mitochondrial genes differentially expressed after PD-1 ligation. (PDF 560 kb) [file 40425_2019_628_MOESM11_ESM.pdf]

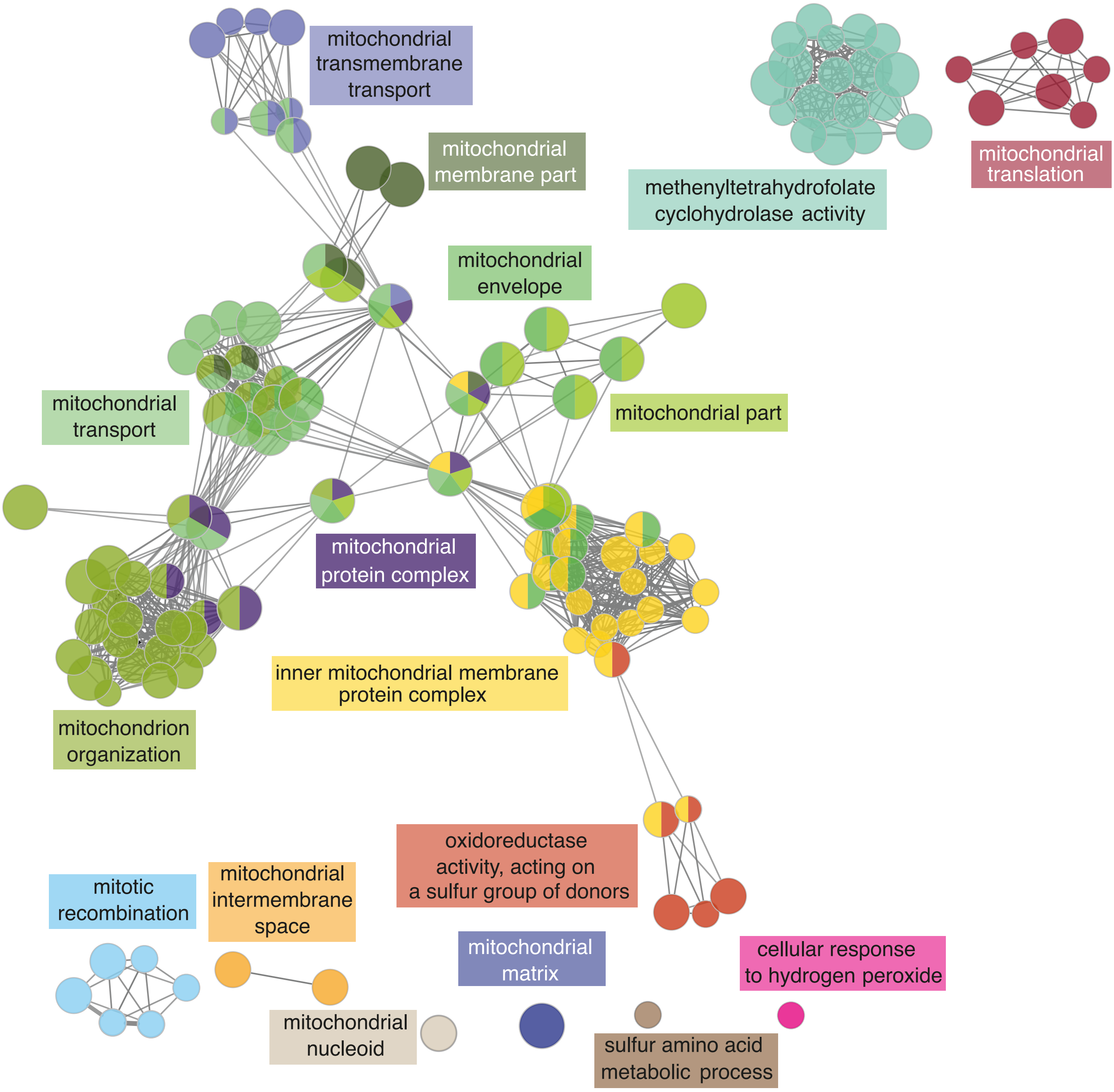

Figure S7. PD-1 targets different mitochondrial pathways and GO categories. ClueGo plot of the 84 mitochondrial genes differentially expressed in T_{ACT} and $T_{ACT+PD1}$ cells. ClueGO visualizes the selected terms in a functionally grouped annotation network that reflects the relationships between the terms based on the similarity of their associated genes. The size of the nodes reflects the statistical significance of the terms. The group leading term for each cluster is indicated.
