# Supplementary material for: PD-1 signaling affects cristae morphology and leads to mitochondrial dysfunction in human CD8+ T lymphocytes
Source: J Immunother Cancer. 2019 Jun 13;7:151. doi: 10.1186/s40425-019-0628-7 (PMC6567413; doi:10.1186/s40425-019-0628-7)
Supplement: Supplementary file 12 — Table S4. List of genes that partition or associate with mitochondria. (PDF 94 kb) [file 40425_2019_628_MOESM12_ESM.pdf]

Table S4. Genes that partition or associate to mitochondrion

Gene	GO Molecular Function	GO Biological Process
ACADS	Acyl-CoA dehydrogenase activity	Cellular component organization<br>Lipid metabolic process
ACOT7	Acyl-CoA hydrolase	Coenzyme A biosynthetic process<br>Lipid metabolic process
ADHFE1	Alcohol dehydrogenase (NAD) activity	Molecular hydrogen transport
ADO	Cysteamine dioxygenase activity	Oxidation-reduction process
ATP5G1	Hydroxylase<br>Lipid binding<br>Transporter	Respiratory electron transport (Complex V)<br>Mitochondrial protein import
ATP5J	Hydroxylase<br>Transporter	Respiratory electron transport (Complex V)
BCAT1	Branched-chain-amino-acid transaminase	Branched-chain amino acid biosynthetic/catabolic processes
BRCA1	Damaged DNA binding<br>Chaperone-like protein	Cellular response to DNA damage<br>Cell cycle
C1QBP	RNA binding. Transcription<br>Carbohydrate derivative binding	Immune system response to stimulus.<br>Mature ribosome assembly
CHCHD3	Protein complex scaffold.<br>Transcription	Cellular component organization (cristae formation; mitochondrial fusion)
CHCHD10	--	Cellular component organization (cristae morphology maintenance). Oxidative phosphorylation
CLPP	Hydrolase	Protein metabolic process
COMT	Transferase	Catecholamine metabolic process
COX8A	Cytochrome-c oxidase activity	Respiratory electron transport (complex IV)
COX18	Transporter	Respiratory electron transport (complex IV assembly)
CYP1B1	Aromatase activity<br>Iron ion binding	Lipid metabolic process<br>Xenobiotic metabolic process
DBI	Receptor binding<br>Lipid binding	Lipid metabolic process<br>Signaling
DCAF5	---	CUL4-DDB1 E3 ubiquitin-protein ligase complex
DNA2	Hydrolase<br>DNA binding	Response to stimulus (mitochondrial DNA repair/replication)
DNAJC5	ATP-dependent protein binding<br>Glycoprotein binding	Cell death (negative regulator)
DTD1	Aminoacyl-tRNA editing activity	tRNA metabolic process<br>DNA replication
DUT	dUTP diphosphatase activity<br>RNA binding	dUTP catabolism<br>Regulation of protein heteromerization
E2F1	DNA binding<br>Transcription	Cell death<br>Cell cycle
FANCG	--	Response to stimulus<br>Mitochondrion organization
FEN1	5’-3’ exonuclease activity<br>DNA binding	Mitochondrial DNA repair<br>DNA replication
FUNDC2	--	Mitophagy
GAPDH	Glyceraldehyde-3-phosphate	Carbohydrate metabolic process

	dehydrogenase	
GARS	Aminoacyl-tRNA ligase activity	Glycy.tRNA aminoacylation
GLRX	Glutathione disulfide oxidoreductase	Cell redox homeostasis
GSTZ1	Transferase<br>Catalytic activity	Aromatic amino acid family metabolic proce
GZMB	Hydrolase	Immune system response to stimulus
HCLS1	Cytoskeletal binding protein	Response to cytokine stimulus<br>Actin filament polarization
HSPA9	Heat shock protein binding	Protein folding (Minos/Mitos complex)
HSPE1	Chaperone binding	Chaperone-mediated protein folding cofacto
HTT	Protein binding	Mitochondrion organization Mitochondrial transport
JMJD7-PLA2G4B	Hydrolase	Lipid metabolic process
MIEF1	Nucleotide binding	Positive regulation of mitochondrial fission
MRPL39	Nucleotide binding	Mitochondrial genome maintenance.<br>Translation
MRPS12	Structural constituent of ribosome	Mitochondrial translation
MRPS33	Structural constituent of ribosome	Mitochondrial translation
MRPS34	Structural constituent of ribosome	Mitochondrial translation
MTCH1	--	Potential mitochondrial transporter<br>Apoptosis
MTHFD1	Ligase. Hydrolase. Oxidoreductase	One-carbon metabolic process
MTHFD1L	Ligase. Hydrolase. Oxidoreductase	One-carbon metabolic process
MTHFD2	Hydrolase. Oxidoreductase	One-carbon metabolic process
MYCBP	Transcription	Regulation of transcription
NDFIP2	Signal transducer activity	Metal ion transport<br>Positive regulation of protein ubiquitination
NDUFAB1	Acyl binding	Fatty acid biosynthetic process<br>Non-catalytic subunit of the mitochondrial membrane respiratory chain (Complex I)
NDUFB3	NADH dehydrogenase (ubiquinone) activity	Respiratory electron transport (Complex I)
NDUFB7	NADH dehydrogenase (ubiquinone) activity	Respiratory electron transport (Complex I)
NME1	Hydrolase<br>Kinase activity	Cell differentiation<br>GTP/CTP/UTP biosynthetic process
NOP58	RNA binding	Ribosome biogenesis<br>SnoRNA localization
NR4A1	DNA binding<br>Protein binding	Cell death<br>Transcription
OCIAD1	--	--
PET100	Unfolded protein binding	Mitochondrial respiratory chain assembly (Complex IV)
PHB	Complement component C3a binding<br>Transcription	DNA biosynthetic process<br>Mitochondrion organization
PLA2G6	Hydrolase<br>ATP-dependent protein binding	Lipid catabolic process<br>Positive regulation of cytochrome c release from mitochondria
POP7	Hydrolase<br>RNA binding	Mitochondrial DNA transcription<br>tRNA processing

PPIF	Isomerase activity<br>Protein binding	Apoptotic mitochondrial changes<br>Regulation of mitochondrial membrane permeability<br>Negative regulation of ATPase activity
PRDX3	Antioxidant activity<br>Oxidoreductase	Cell redox homeostasis<br>Response to oxidative stress
RPUSD4	Pseudouridine synthase activity	Pseudouridine synthesis<br>RNA modification
SCCPDH	Oxidoreductase	Oxidation/reduction process
SELRC1	--	Assembly of mitochondrial respiratory chai[illegible]<br>(Complex I & IV)
SFXN1	Ion transporter	Mitochondrial iron ion transport
SHMT2	Amino acid binding<br>Chromatin binding	Glycine/serine metabolism<br>One-carbon metabolic process
SLC9A6	K+/Na+:proton antiporter activity	Calcium homeostasis
SLC16A1	Monocarboxylic acid and lactate transmembrane transporter activity	Glucose homeostasis<br>Lipid metabolic process
SLC25A19	Mitochondrial thiamine pyrophosphate transporter activity	Transmembrane transport
SLC25A45	Mitochondrial transporter activity	Transmembrane transport
SP1	Transcription	Cellular response to insulin stimulus
TAOK3	Serine/Threonine kinase	Positive regulation of stress-regulated MAP[illegible] cascade<br>DNA repair
TIMM22	Mitochondrial protein import and insertion of transmembrane proteins	Protein import into mitochondrial inner membrane
TIMM23	Transporter (protein channel activity)	Protein import into mitochondrial matrix
TOMM34	Heat shock protein binding	Protein targeting into mitochondrion
TOMM40	Porin activity	Protein import into mitochondrial matrix
TOMM40L	Porin activity	Protein import into mitochondrial matrix
TOP2A	DNA topoisomerase type II (ATP hydrolyzing) activity	Cell death. DNA ligation. DNA repair. Posit[illegible] regulation of transcription
TXN1	Oxidoreductase. RNA binding	Redox homeostasis
TXN2	Oxidoreductase	Redox homeostasis.<br>Glycerol ether metabolic process
UQCRC2	Metalloendopeptidase activity	Oxidation-reduction process<br>Protein processing involved in protein target[illegible] to mitochondrion
VDAC1	Transporter<br>Porin activity	Mitochondrial calcium ion transport.<br>Regulation of mitophagy
XRCC3	DNA binding	DNA recombination and repair
YWHAE	Protein binding	Cellular response to heat<br>Negative regulation of protein dephosphorylation
YWHAH	Cytoskeletal protein binding<br>Receptor binding	Cytoskeleton organization<br>Glucocorticoid receptor signaling
