# Supplementary material for: PD-1 signaling affects cristae morphology and leads to mitochondrial dysfunction in human CD8+ T lymphocytes
Source: J Immunother Cancer. 2019 Jun 13;7:151. doi: 10.1186/s40425-019-0628-7 (PMC6567413; doi:10.1186/s40425-019-0628-7)
Supplement: Supplementary file 13 — Table S5. GO enrichment analysis of profile B by STEM (top 20). (PDF 84 kb) [file 40425_2019_628_MOESM13_ESM.pdf]

**Table S5. GO enrichment analysis of profile B by STEM (top 20)**

| Category ID | Category name | $N_{cat}$ | $N_{allo}$ | $N_{exp}$ | $N_{enr}$ | p-val | $p\text{-val}_{Adj}$ | F |
|---|---|---|---|---|---|---|---|---|
| GO:0045177 | Apical part of the cell | 9 | 5 | 1.2 | 3.8 | $2.7 \times 10^{-3}$ | 0.514 | 4.3 |
| GO:0004872 | Receptor activity | 27 | 9 | 3.5 | 5.5 | $4.3 \times 10^{-3}$ | 0.624 | 2.6 |
| GO:0004888 | Transmembrane signaling receptor activity | 15 | 6 | 1.9 | 4.1 | $7.5 \times 10^{-3}$ | 0.802 | 3.1 |
| GO:0099600 | Transmembrane receptor activity | 15 | 6 | 1.9 | 4.1 | $7.5 \times 10^{-3}$ | 0.802 | 3.1 |
| GO:0005509 | Calcium ion binding | 13 | 5 | 1.7 | 3.3 | 0.02 | 0.956 | 3.0 |
| GO:0043169 | Cation binding | 115 | 22 | 14.9 | 7.1 | 0.02 | 0.968 | 1.5 |
| GO:0046872 | Metal ion binding | 115 | 22 | 14.9 | 7.1 | 0.02 | 0.968 | 1.5 |
| GO:0001701 | *In utero* embryonic development | 19 | 6 | 2.5 | 3.5 | 0.03 | 0.982 | 2.4 |
| GO:0098590 | Plasma membrane region | 19 | 6 | 2.5 | 3.5 | 0.03 | 0.982 | 2.4 |
| GO:0031224 | Intrinsic component of membrane | 123 | 23 | 16 | 7 | 0.03 | 0.982 | 1.4 |
| GO:0016021 | Integral component of membrane | 117 | 22 | 15.2 | 6.8 | 0.03 | 0.982 | 1.4 |
| GO:0038023 | Signaling receptor activity | 21 | 6 | 2.7 | 3.3 | 0.04 | 0.99 | 2.2 |
| GO:0043167 | Ion binding | 185 | 31 | 24 | 7 | 0.04 | 0.992 | 1.3 |
| GO:0001503 | Ossification | 16 | 5 | 2.1 | 2.9 | 0.04 | 0.992 | 2.4 |
| GO:0043410 | Positive regulation of MAPK cascade | 17 | 5 | 2.2 | 2.8 | 0.06 | 0.996 | 2.3 |
| GO:0000165 | MAPK cascade | 29 | 7 | 3.8 | 3.2 | 0.07 | 0.998 | 1.9 |
| GO:0023014 | Signal transduction by protein phosphorylation | 29 | 7 | 3.8 | 3.2 | 0.07 | 0.998 | 1.9 |
| GO:0007166 | Cell surface receptor signaling pathway | 73 | 14 | 9.5 | 4.5 | 0.07 | 0.998 | 1.5 |
| GO:0044425 | Membrane part | 152 | 25 | 19.7 | 5.3 | 0.09 | 1 | 1.3 |

$N_{cat}$, number of annotated genes to a GO category; $N_{allo}$, number of genes of a GO category that were allocated to Profile 42; $N_{exp}$, number of genes of a GO category expected to be assigned to this gene set [(Genes_In_Set)*(Genes_Category)/(Total_Unique_Genes)]; $N_{enr}$, difference between $N_{allo}$ and $N_{exp}$; p-val, uncorrected probability of genes assigned *vs* expected; $p\text{-val}_{Adj}$, p-value corrected for multiple GO categories tested simultaneously; F, fold of enrichment ($N_{allo}/N_{exp}$).
