# Supplementary material for: PD-1 signaling affects cristae morphology and leads to mitochondrial dysfunction in human CD8+ T lymphocytes
Source: J Immunother Cancer. 2019 Jun 13;7:151. doi: 10.1186/s40425-019-0628-7 (PMC6567413; doi:10.1186/s40425-019-0628-7)
Supplement: Supplementary file 14 — Figure S8. Changes in mitochondria-related gene expression is PD-L1 dose-dependent. (PDF 166 kb) [file 40425_2019_628_MOESM14_ESM.pdf]

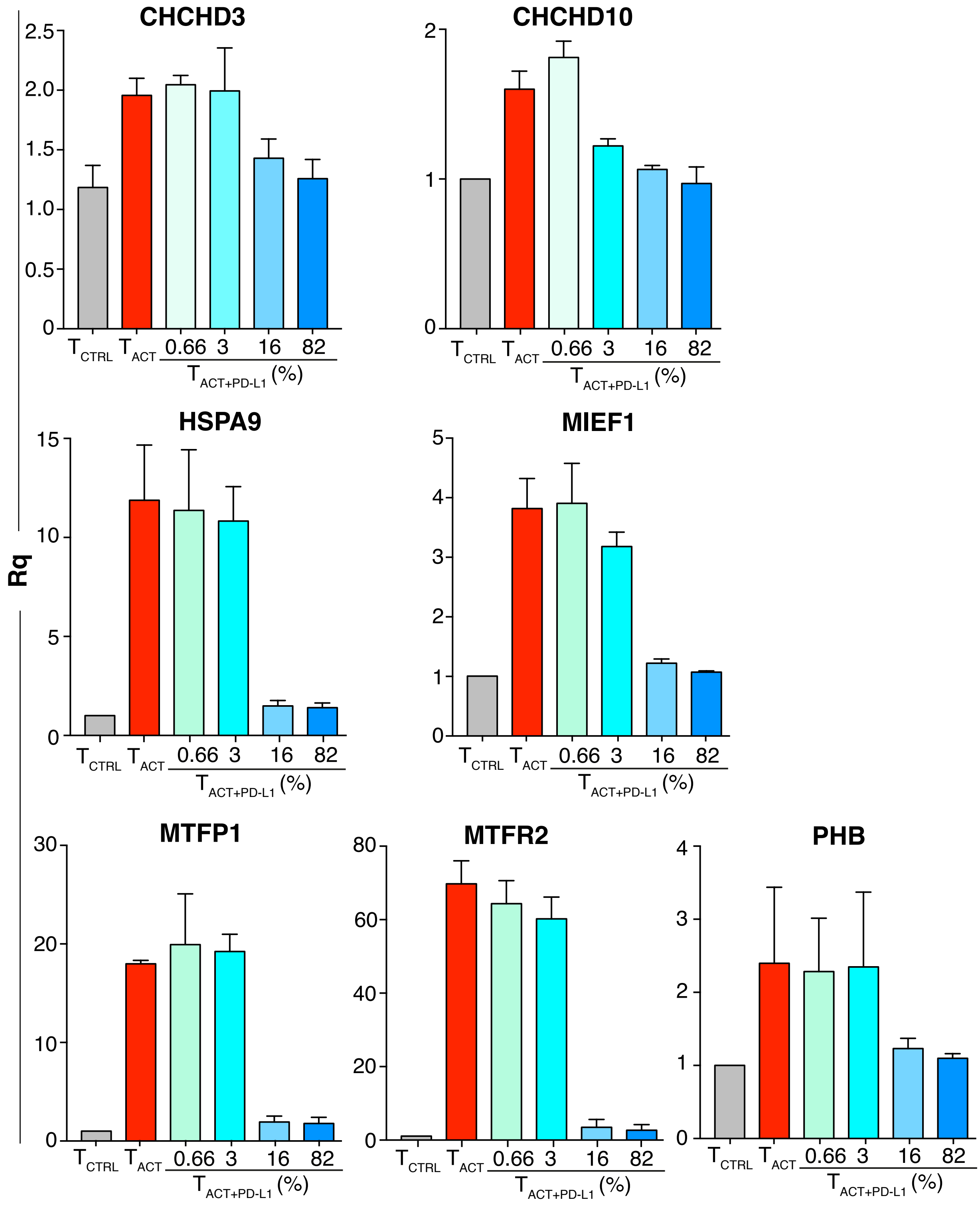

Figure S8. Changes in mitochondrial-related gene expression are PD-L1 dose-dependent. Purified human CD8+ T cells were incubated (48 h) with T_{CTRL}, T_{ACT}, or $T_{ACT+PD1}$ beads containing the indicated amounts of PD-L1-Fc. Variation of the relative quantity (Rq) of indicated transcripts was determined by qPCR. Data are mean ± SD from two donors.
