# Supplementary material for: PD-1 signaling affects cristae morphology and leads to mitochondrial dysfunction in human CD8+ T lymphocytes
Source: J Immunother Cancer. 2019 Jun 13;7:151. doi: 10.1186/s40425-019-0628-7 (PMC6567413; doi:10.1186/s40425-019-0628-7)
Supplement: Supplementary file 15 — Figure S9. Mitochondrial morphology analyzed by TEM. (PDF 5455 kb) [file 40425_2019_628_MOESM15_ESM.pdf]

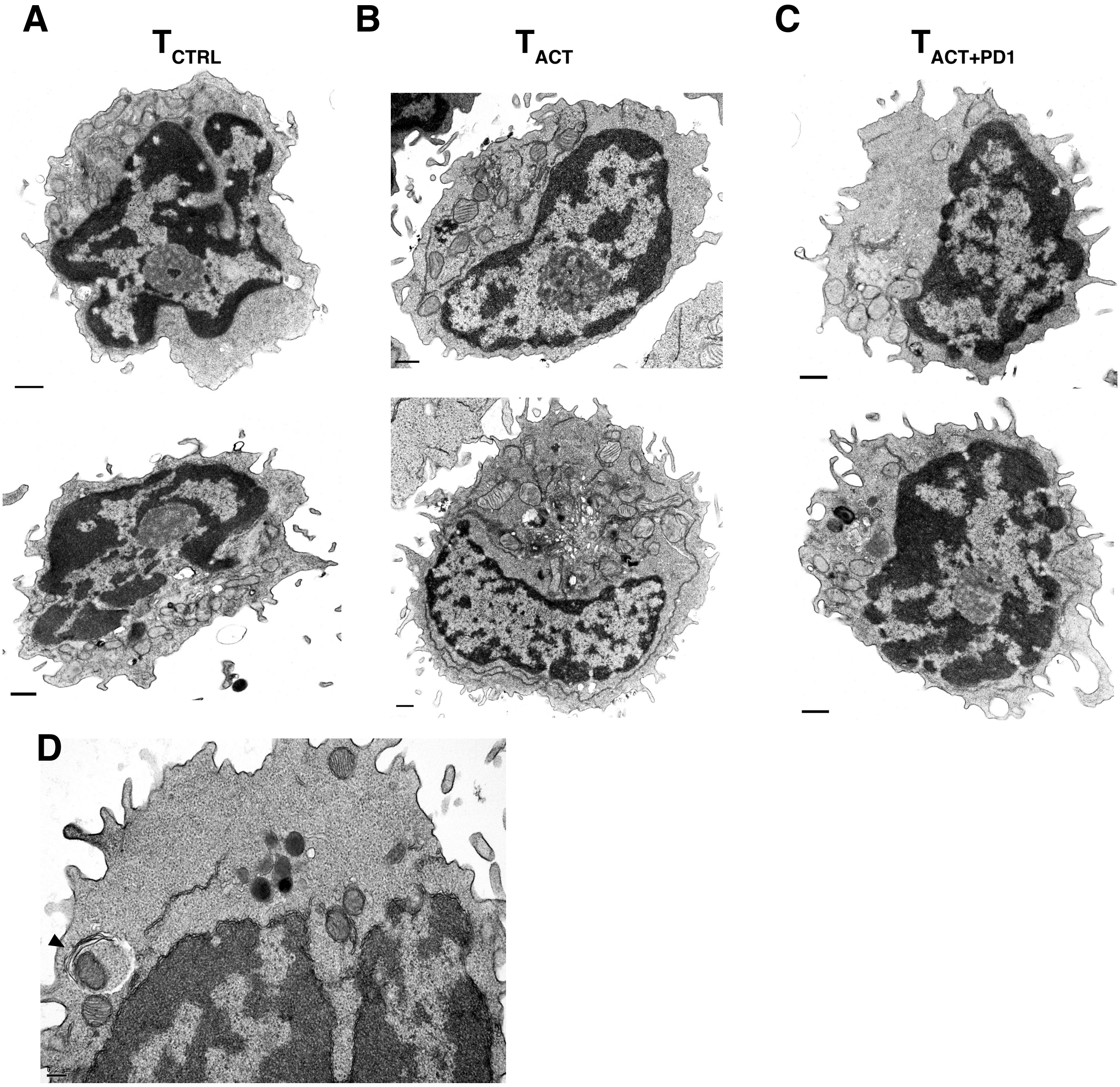

Figure S9. Mitochondrial morphology analyzed by TEM. (**A-C**) Two representative TEM images from T_{CTRL} (*A*), T_{ACT} (*B*) and $T_{ACT+PD1}$ cells (*C*). Bar, 0.5µm. (**D**) A representative TEM image from a $T_{ACT+PD1}$ cell, with a structure resembling an autophagosome (arrowhead). Bar, 0.2µm (bottom left).
